# Selective Solid–Liquid Extraction of Lithium Cation Using Tripodal Sulfate-Binding Receptors Driven by Electrostatic Interactions

**DOI:** 10.3390/molecules29112445

**Published:** 2024-05-22

**Authors:** Ya-Zhi Chen, Ying-Chun He, Li Yan, Wei Zhao, Biao Wu

**Affiliations:** 1 Key Laboratory of Medicinal Molecule Science and Pharmaceutics Engineering, Ministry of Industry and Information Technology, School of Chemistry and Chemical Engineering, Beijing Institute of Technology, Beijing 102488, China; 2 Institute of Applied Chemistry, Shanxi University, Taiyuan 030006, China; 3 Analysis & Testing Center, Beijing Institute of Technology, Beijing 102488, China

**Keywords:** anion receptor, lithium separation, molecular recognition, oligourea, solid–liquid extraction, supramolecular chemistry

## Abstract

Owing to the important role of and increasing demand for lithium resources, lithium extraction is crucial. The use of molecular extractants is a promising strategy for selective lithium recovery, in which the interaction between lithium and the designed extractant can be manipulated at the molecular level. Herein, we demonstrate that anion receptors of tripodal hexaureas can selectively extract Li_2_SO_4_ solids into water containing DMSO (0.8% water) compared to other alkali metal sulfates. The hexaurea receptor with terminal hexyl chains displays the best Li^+^ extraction selectivity at 2-fold over Na^+^ and 12.5-fold over K^+^. The driving force underpinning selective lithium extraction is due to the combined interactions of Li^+^-SO_4_^2−^ electrostatics and the ion–dipole interaction of the lithium–receptor (carbonyl groups and N atoms); the latter was found to be cation size dependent, as supported by computational calculations. This work indicates that anion binding receptors could drive selective cation extraction, thus providing new insights into the design of receptors for ion recognition and separation.

## 1. Introduction

Lithium, the “white gold” for the energy transition, is deemed as a critical ingredient in powering renewable energy storage systems and electric vehicles, specifically in lithium-ion batteries (LIBs) [1]. As the demand for lithium-ion batteries has dramatically increased in recent years (150,000–190,000 tons by 2030) [2], utilizing lithium production to meet the market and sustainability targets in the future will be challenging [3]. Thus, in addition to conventional lithium production methods of evaporative brine processing and ore mining [4], developing new lithium extraction methods is crucial [5]. Currently, emerging approaches include adsorption [6], ion exchange [7], and solvent extraction [8]. In contrast, given the challenges of selective lithium extraction in the presence of other alkali cations and alkali earth metal ions [9], solvent extraction (including liquid–liquid extraction (LLE) and solid–liquid extraction (SLE)) is thought to be promising in handling this challenge by extracting lithium selectively from water or solids into organic solvents using molecular extractants.

Molecular extractants can be engineered with tailored binding motifs to facilitate selective lithium binding and extraction at the molecular level [10]. In the supramolecular chemistry field, reported extractants normally include cation-binding receptors and ion-pair receptors [11]. A pioneering lithium binding receptor was reported in the 1980s, displaying selective lithium extraction using spherand macrocycles [12,13], followed by many crown ether macrocycles and cryptand-based receptors [14,15,16]. These systems typically rely on an ion exchange process or are suitable for use in the presence of lipophilic countercations (e.g., picrate and perchlorate) for the extraction process [17]. Through complexation with cation binding receptors, the physical and chemical properties of initial salts will change accordingly [18], i.e., the studied cation will be decorated with an “organic skin” (receptor) thus influencing the cation–solvents and cation–anion interactions and changing their solubilities. The counter-anion could also be activated as a relatively “naked” anion for the desired purpose, like the process seen in the phase-transfer catalysis reaction. Later, ion-pair receptors concurrently encoded with cation binding motifs and anion binding motifs were developed [19,20,21]. Typical examples include strapped crown ether and calix [4] pyrrole-based macrocycles, which have been demonstrated to selectively separate lithium from other alkali cations via solvent extraction [22,23,24,25]. It is noted that ion-pair receptors mostly rely on sophisticated chemical synthesis, thus limiting their real applications. Intuitively, it is thought to be not feasible to extract cations without the help of cation-binding motifs.

**Figure 1:**
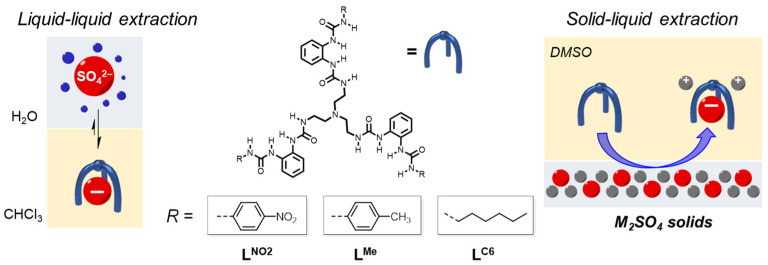
Liquid–liquid extraction of sulfate in previous studies and the solid–liquid extraction of M_2_SO_4_ solids in this work by using tripodal hexaurea receptors.

Through comparison with the above-mentioned approaches, another viable way is to use binary mixtures of anion receptors and cation receptors for extraction by avoiding any possible challenge that may come up upon synthesizing delicate ion-pair receptors. It has been clearly demonstrated that an anion can be selectively bound or extracted by using designed receptors with specific sizes and shapes and incorporating anion bonding sites (particularly using hydrogen bonding) [26,27,28,29,30]. In this work, the original idea was to use commercially available crown ether macrocycles and previously prepared oligourea receptors for alkali salt separation. To our surprise, tripodal hexaurea receptors alone can selectively extract Li_2_SO_4_ solids into DMSO (with 0.8% water, Figure 1). As indicated in previous studies, tripodal hexaurea receptors display strong and selective sulfate binding (over 10^5^ M^−1^ in DMSO) thus enabling nearly quantitative sulfate extraction and pH-dependent release [31,32,33,34]. Here, upon alkali metal cation extraction, strong sulfate binding in combination with electrostatic interactions between receptor-complexed sulfate and alkali cations can further drive the selective uptake of lithium via solid–liquid extraction. Density function theory (DFT) calculations indicate that extraction selectivity is possibly governed by sulfate–cation electrostatic interaction and the size of alkali cations.

## 2. Results and Discussion

According to our previous studies, tripodal hexaurea receptors bind to sulfate with dependence upon terminal substitutions (**L^NO2^**, **L^Me^**, and **L^C6^**, Figure 1), which can regulate their hydrogen bonding strength upon anion binding. These free hexaurea receptors were synthesized based on previously reported procedures, and the structures were characterized and confirmed via ^1^H NMR (Supplementary Materials). In order to extract alkali metal sulfate salts of M_2_SO_4_ (M = Li, Na, K, Rb, Cs), we used a binary mixture of 18-crown-6 ether (for cation binding) and oligourea receptors (for anion binding) in solid–liquid extraction. DMSO was used as the organic solvent because the hexaurea receptor could only solubilize in a polar solvent, and the solubility of M_2_SO_4_ solids in regular DMSO is negligible (<0.2 mM, vide infra). The receptor **L^NO2^** was first tested because of its stronger sulfate binding affinity than other receptors. By mixing M_2_SO_4_ solids with one equivalent of **L^NO2^** and two equivalents of 18-crown-6 ether in DMSO at room temperature for a few hours (Supporting Information, SI, Table S8), clear solutions were obtained for Li_2_SO_4_ and Na_2_SO_4_, indicating efficient solid–liquid extraction. In contrast, there were still undissolved solids for K_2_SO_4_, Rb_2_SO_4_, and Cs_2_SO_4_. The ^1^H NMR and ion chromatography (IC) results suggested that the extraction efficiency (defined as solubilized cations versus the initial amount of alkali cations) followed the order of Li^+^ > Na^+^ > Cs^+^ > Rb^+^ > K^+^. Interestingly, in the presence of the **L^NO2^** receptor alone (without adding 18-crown-6 ether for cation binding), very similar extraction efficiency was also observed for all of the M_2_SO_4_ solids, indicating that crown ether did not help to solubilize the alkali cation in DMSO. This is presumably because the binding affinities of 18-crown-6 ether with alkali cations are too weak in the highly polar solvent, DMSO (dielectric constant = 46.7), even for Na^+^ and K^+^ cations [35]. It was noted that the anion receptor **L^NO2^** alone can fully drive the extraction of M_2_SO_4_ with Li^+^ selectivity. To the best of our knowledge, it is unusual for anion receptors to selectively extract M_2_SO_4_ solids into DMSO via LLE, in which electrostatic interactions between receptor-complexed sulfate and alkali cation are the driving force.

**Figure 2:**
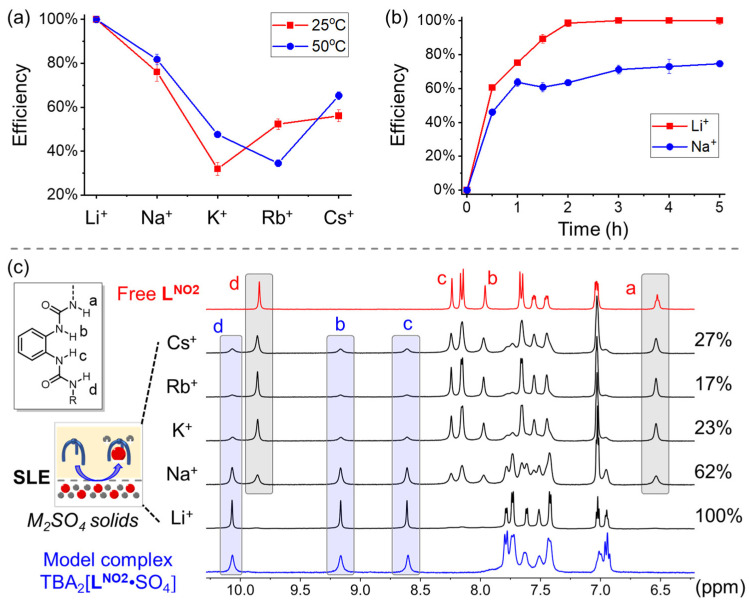
(**a**) Temperature-dependent and (**b**) time-dependent extraction efficiency of alkali cations obtained from SLE by using the receptor **L^NO2^** (in DMSO with 0.8% water). Efficiency was recorded via ion chromatography. (**c**) Stacked ^1^H NMR spectra (1 mM, 400 MHz, DMSO-*d*_6_, 298 K) after SLE indicating selective lithium extraction. The obtained extraction efficiency is shown on the right derived from ion chromatography.

To improve lithium extraction selectivity, the condition used for solid–liquid extraction was screened by varying the temperature and processing time. Specifically, by stirring the SLE mixture at 25 °C and 50 °C for 5 h, the same extraction efficiency was seen for Li^+^, indicating quantitative lithium extraction (Figure 2a). The extraction efficiency of Na_2_SO_4_, K_2_SO_4_, and Cs_2_SO_4_ increased simultaneously with the increasing temperature, while that of Rb_2_SO_4_ was found to decrease. It was speculated that heating would lead to a clear decrease in the binding affinity between **L^NO2^** and Rb_2_SO_4_. The lithium extraction selectivity over the K^+^ cation decreased from 3.3-fold to 2.0-fold as the temperature increased from 25 °C to 50 °C, suggesting favorable extraction selectivity for Li_2_SO_4_ at low temperatures. By recording the extraction over time at 25 °C (Figure 2b), the extraction efficiency significantly improved in the first hour and was completed in 2 h as indicated by the ^1^H NMR and IC results. In contrast, only 62% of Na_2_SO_4_ was extracted in 2 h. Additionally, the solid–liquid extraction kinetics were also studied. By changing the stirring rate from 500 r/min to 1500 r/min (25 °C and 1 h, Table S11), the extraction efficiencies for Li_2_SO_4_ and Na_2_SO_4_ were found to slightly increase by 8% (65% to 73%) and 6% (58% to 64%), respectively. Therefore, the optimal SLE condition for selective lithium extraction was found—temperature: 25 °C, time: 2 h, and stirring rate: 1500 r/min, which were all used for the following SLE experiments. Under the same SLE conditions, without adding a receptor, the M_2_SO_4_ salts alone do not dissolve in DMSO with 0.8% water.

In contrast, under the same SEL conditions (25 °C, 2 h, 1500 r/min), the extraction efficiencies of K_2_SO_4_, Rb_2_SO_4_, and Cs_2_SO_4_ by using the receptor **L^NO2^** were determined to be 23%, 17%, and 27%, respectively (Table 1). Based on the ^1^H NMR spectra (Figure 2c), the species of the free receptor and sulfate-complexed receptor can be clearly assigned. Through comparison with the ^1^H NMR spectrum of the model complex of TBA_2_[**L^NO2^**·SO_4_] (TBA: tetrabutylammonium), all of the receptors were complexed with Li_2_SO_4_, indicating quantitative and selective lithium extraction, consistent with the IC data. The determined lithium extraction efficiency was approximately 1.6-fold over Na^+^, 4.3-fold over K^+^, 5.9-fold over Rb^+^, and 3.7-fold over Cs^+^. The overall extraction efficiency of **L^NO2^** follows the order: Li_2_SO_4_ > Na_2_SO_4_ > Cs_2_SO_4_ > Rb_2_SO_4_ > K_2_SO_4_.

By changing the receptor **L^NO2^** to the other two hexaurea receptors, **L^Me^** (with terminal 4-methylphenyl, Figure 1) and **L^C6^** (with terminal hexyl chains), similar results for extraction efficiency and selectivity were observed (Table 1). Specifically, all three hexaurea receptors displayed quantitative lithium extraction (100%). For Na_2_SO_4_ and K_2_SO_4_, the extraction efficiency followed the order of **L^NO2^** > **L^Me^** > **L^C6^**, which is consistent with their sulfate binding affinity (normalized sulfate binding affinity: **L^NO2^**:**L^Me^**:**L^C6^** = 6.7:1.7:1) [33]. Relatively higher lithium extraction selectivity was observed for the receptor **L^C6^**, which was attributed to its relatively weak sulfate binding, thus allowing for relatively tight attraction between receptor-complexed sulfate and lithium cations as supported by DFT calculations (vide infra).

Solid–liquid extraction was also conducted by using a mixture of 12-crown-4 ether and the **L^C6^** receptor to see if cation binding could promote lithium extraction. Under stirring in DMSO with 0.8% water for one hour at 25 °C, we observed very similar lithium extraction efficiency by using the **L^C6^** receptor alone (87%, Figure S31) or by using the mixture of the **L^C6^** receptor and 12-crown-4 ether (91%). This indicates that the cation binding is too weak to promote extraction in DMSO, and the observed extraction may be mainly driven by electrostatic interactions between receptor-complexed sulfate and cations. We performed SLE for the MgSO_4_ solids as well, using the same conditions, showing that all of the MgSO_4_ solids could be dissolved in DMSO (Figure S32). This is presumably due to the strong electrostatic interaction between Mg^2+^ and receptor-complexed sulfate. 

A tripodal triurea receptor, **TL^C6^**, with the same terminal substitution of hexyl chains was also studied [19]. Clearly lower alkali metal cation extraction efficiency was seen by comparing it to that of **L^C6^** (Table 1). This is because a 2:1 sandwich complex of triurea receptors and sulfate would typically form, thus consequently restricting the electrostatic interaction between complexed sulfate and alkali cations [18]. In addition, we also attempted to conduct the extraction experiments by changing the counter-anion from sulfate to chloride or acetate. However, all of the MCl and CH_3_COOM solids are soluble in DMSO, thus limiting their use in SLE studies. Overall, all of these results suggest that the characteristic sulfate binding of hexaurea receptors is critical for selective lithium extraction.

**Figure 3:**
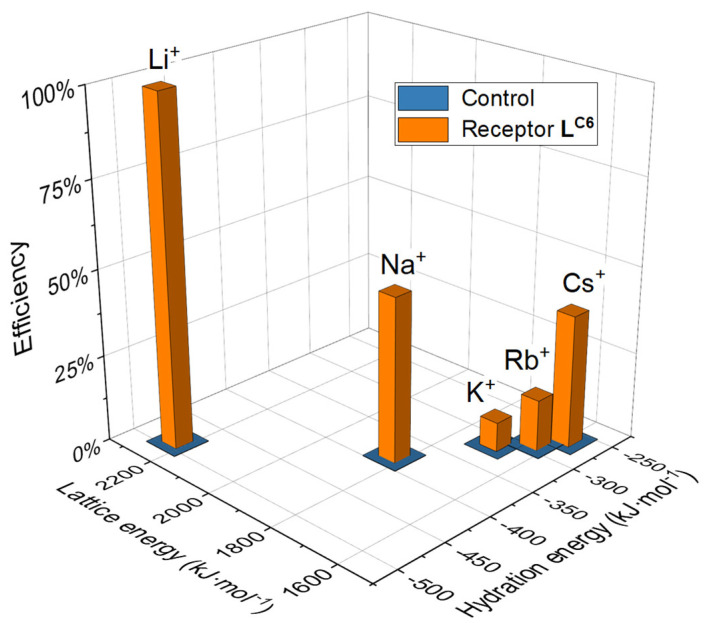
Schematic illustration of efficiency dependence with the polarizability of alkali cations and their lattice energies with sulfate anions. The data are shown by using the receptor **L^C6^** in SLE experiment results. The control indicates that only M_2_SO_4_ solids were used without adding a receptor.

The hexaurea receptor with relatively weaker sulfate binding than the other two hexaurea receptors displays the best lithium extraction selectivity, the extraction efficiency of alkali metal sulfates follows the order of Li_2_SO_4_ > Na_2_SO_4_ > Cs_2_SO_4_ > Rb_2_SO_4_ > K_2_SO_4_. The observed lithium extraction efficiency is 2.0-fold over Na^+^, 12.5-fold over K^+^, 6.7-fold over Rb^+^, and 2.6-fold over Cs^+^. Most likely, the alkali metal sulfates were transferred into organic solvent in the form of associated ion pairs upon the solid–liquid extraction process. Thus, the extraction efficiency and selectivity would be dependent on the electrostatic interactions between receptor-complexed sulfate and alkali cations, which is related to two conflicting factors: alkali metal cations’ hydration energy and their corresponding lattice energies with sulfate (Figure 3).

First, according to the lattice energy of the sulfate and alkali cation, the electrostatic attraction between sulfate and lithium would be the strongest. Although the complexation from the hexaurea receptor would more or less inhibit the interaction between sulfate and the environment, all of the electrostatic interactions with various alkali cations would decrease to a very similar extent. Therefore, the best extraction efficiency is expected for lithium cations. On the other hand, according to hydration energy, the cation with the largest hydration energy is thought to be the most difficult to extract. As hydration decreases, the cesium cation is expected to extract the most. As a result, we observed the best extraction for Li+ (100%) and the smallest extraction for K^+^ (8%) by using the receptor **L^C6^** (Figure 3). Therefore, the observed overall extraction efficiency is believed to be governed by electrostatic interactions and alkali cations’ hydration properties.

**Figure 4:**
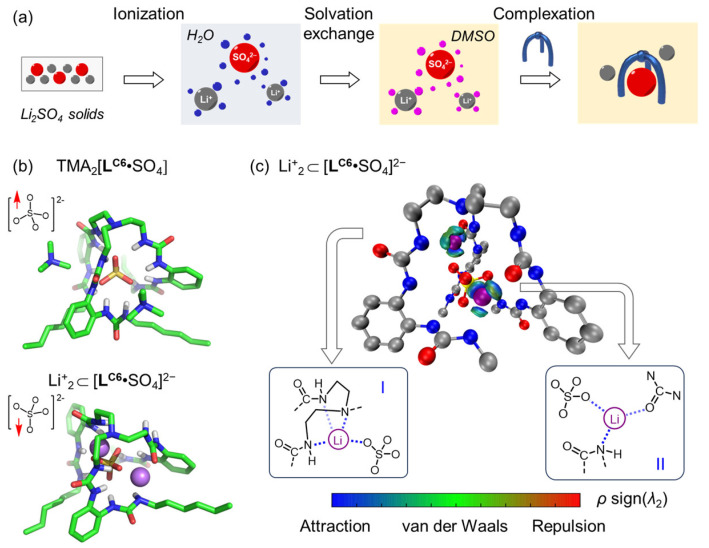
(**a**) Proposed mechanism underpinning solid–liquid extraction. DFT-optimized structures for sulfate-binding complexes of the receptor **L^C6^** with (**a**) tetramethylammonium (TMA) and (**b**) lithium counter-cations at the theory level of B3LYP/6-31G(D). (**c**) IGM plot of Li^+^_2_ ⊂ [**L^C6^**•SO_4_]^2−^ illustrating that two lithium cations are stabilized by attraction with sulfate anions, N and O=C moieties. Color coding in the range of −0.5 < *ρ* sign(*λ*_2_) < 0.5 a.u. [37,38]. Atom colors: green or grey = C, blue = N, red = O, yellow = S, and purple = Li.

To figure out the reason why lithium is selectively extracted, we deconvoluted the process involved in the entire solid–liquid process (Figure 4a). It is proposed that a three-step SLE process may occur, and a trace amount of water (16 μL, 0.8%) is critical to ionize Li_2_SO_4_ solids and to initialize the next two steps of solvation exchange and complexation. Firstly, the Li^+^ and SO_4_^2−^ are hydrated and partially separated by water molecules in the ionization step (i.e., Li_2_SO_4_ in water solution, 625 mM). In the second step, a mass amount of DMSO molecules will replace the water shell (blue spheres) to form DMSO-solvated ions (pink spheres). It has been widely proven that lithium cations can coordinate with four DMSO molecules by forming [Li(DMSO)_4_]^+^ [36]. Finally, as the complexation of sulfate and the receptor occurs, the ion-pairing of receptor-complexed sulfate and lithium will occur. In contrast, by using the pure M_2_SO_4_ solids without adding water (i.e., in pure DMSO), the hexaurea receptors cannot solubilize the solids, suggesting that the first process of ionization in trace amounts of water is critical. Additionally, by not adding hexaurea receptors, the M_2_SO_4_ solids are not dissolved in DMSO with 0.8% water, indicating that complexation with the receptor is indispensable. These results are consistent with the proposed three-step process for solid–liquid extraction. It is also indicated that the hydration strength of ions will affect overall SLE efficiency; it would be favorable for less hydrated cations to be dissolved in DMSO.

To help understand the ion-pair interaction between receptor-complexed sulfate anions and alkalic cations, we attempted to quantify the overall binding affinity of the receptor and Li_2_SO_4_ by using ^1^H and ^7^Li NMR titration. Unfortunately, negligible chemical shift changes were observed in NMR spectra, thus making it difficult to distinguish the overall binding affinities with various alkali metal cations. Therefore, DFT calculations were performed for the structural optimization of the complexation of **L^C6^** with various M_2_SO_4_ salts using Spartan 20 at the theory level of B3LYP/6-31G(D) in DMSO continuum with the Conductor-like Polarizable Continuum Model. The sulfate binding geometry of the **L^C6^** receptor with tetramethylammonium (TMA) countercations was first calculated (Figure 4b), in which the obtained structure (including hydrogen bonding interactions and the relative location of TMA cations) is comparable to the previously reported crystal structure [26], suggesting that the method used for DFT calculations is reliable, and subsequently applied for the calculation with M_2_SO_4_ salts. Specifically, sulfate is stabilized through twelve hydrogen bonds, and one of four O atoms is pointed to the central N atom (see the chemical structure in Figure 4b). Two TMA cations are seen to reside outside the complex, showing a distance of N(TMA)···S(SO_4_) at 5.8 Å.

**Figure 5:**
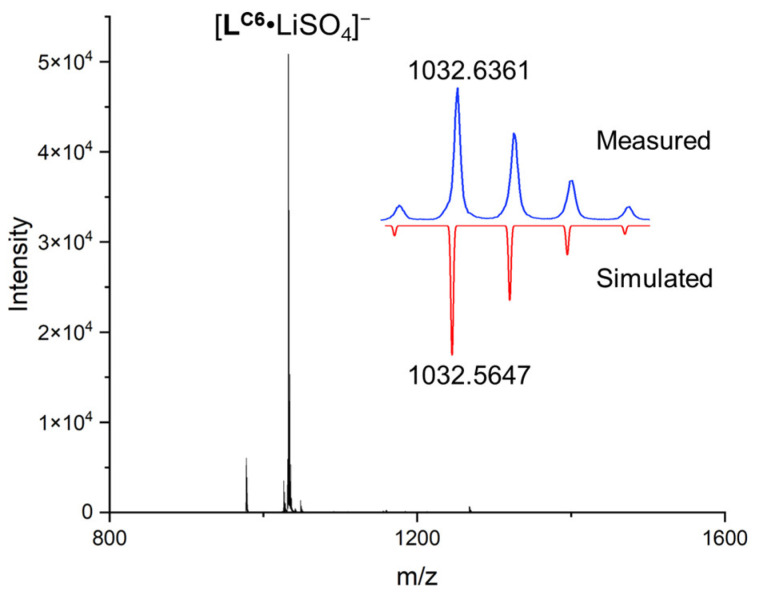
Obtained HR-ESI-QTOF mass spectrum for the complex of **L^C6^** with Li_2_SO_4_.

In contrast, by replacing the two TMA cations with two Li^+^, we observed that the contacts between Li^+^ and sulfate moved much closer to one another, and the sulfate anion was clearly rotated (Figure 4c, see the chemical structure in the right-hand corner). One lithium cation goes into the cavity between the sulfate and the central tris(2-aminoethyl)amine spacer. The independent gradient model (IGM) plot illustrates that lithium is stabilized by four strong attractive interactions with sulfate, the central N atom, and N(H) of urea units (structure I in Figure 4c). The other lithium is seen to be stabilized by three attractive interactions with sulfate, N(H), and the O=C of urea units (structure II in Figure 4c). The average distance among these interactions was found to be 2.08 ± 0.15 Å, indicating strong attraction. Such ion–dipole interaction between alkali cations and carbonyl groups has been observed before in the complex of receptor **L^C6^** with K_2_SO_4_, where the K^+^ cation is also encapsulated inside an 18-crown-6 ether macrocycle in the crystal structure [33]. Notably, the DFT-supported interaction between the lithium and sulfate anion could be considered as second-sphere coordination, which is commonly seen in transition metal complexes and supramolecular complexes [39]. Consistently, such strong complexation can be observed in the mass spectrum. Specifically, a clear peak at 1032.6361 was found and assigned to the complex of [**L^C6^**·LiSO_4_] ^−^ (Figure 5). Overall, the DFT calculations and mass spectrometry data consistently suggest a strong interaction between receptor-complexed sulfate and lithium cations.

Similar structures and attractive interactions were also seen for the complex with Na_2_SO_4_ with a relatively longer attractive distance (2.4 ± 0.1 Å) of Na^+^ (ion radius 1.02 Å) and sulfate, urea units (Figures S8–S10). This indicates relatively weaker Na^+^ binding compared to that of the Li_+_ cation. In contrast, large alkali cations of K^+^ (ion radius 1.38 Å), Rb^+^ (ion radius 1.52 Å), and Cs^+^ (ion radius 1.67 Å) were found to reside outside the complexed sulfate structure, which is similar to the location of TMA cations, suggesting negligible cation attraction with sulfate (Figures S11–S19). All of these computational results are consistent with the extraction data and support relatively stronger lithium interaction with complexed sulfate anions than other cations.

**Figure 6:**
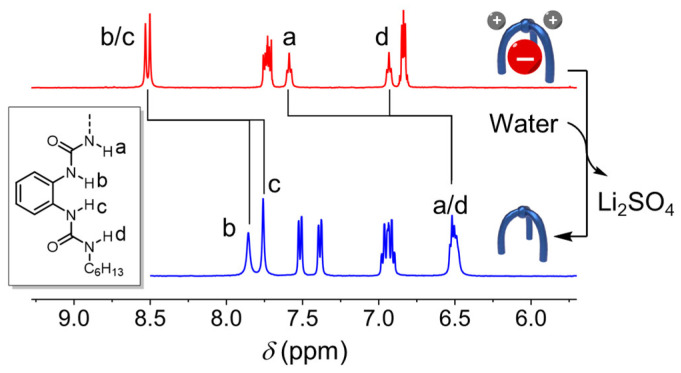
Stacked ^1^H NMR spectra (1 mM, 400 MHz, DMSO-*d*_6_, 298 K) of sulfate complexed with lithium before and after water washing suggesting the release of free receptor **L^C6^** and Li_2_SO_4_.

Furthermore, the extracted lithium sulfate salts can be readily released just by adding an excess amount of water into DMSO. The free receptor precipitates and is recycled. All of the complexed receptors with Li_2_SO_4_ were converted to free receptors as confirmed via ^1^H NMR spectroscopy (Figure 6), where only the peaks assigned to the free receptor were observed. The recycled receptor can be used for the future solid–liquid extraction of alkali metal sulfate solids with retained performance.

## 3. General Synthetic and Solid–Liquid Extraction Procedures

Synthetic procedures: All of the ligands were made beforehand and prepared according to previously reported procedures by using commercially available N^1^,N^1^-bis(2-aminoethyl)ethane-1,2-diamine and corresponding isocyanates. The structures were characterized and confirmed via NMR. Detailed procedures and characterization can be found in the Supporting Information. 

The general solid–liquid extraction procedure: A solution of the receptor (5 mM, 2 mL, DMSO) was exposed to an aqueous solution (16 μL) containing targeted alkali metal cations (e.g., Li_2_SO_4_, Na_2_SO_4_, K_2_SO_4_, Rb_2_SO_4_, and Cs_2_SO_4_, 625 mM). The added M_2_SO_4_ solids were seen to rapidly precipitate. The prepared solution mixture was stirred at 25 °C for 2 h with a stirring rate of 1500 r/min. The solution and undissolved solids were separated upon centrifugation. All of the SLE experiments were repeated three times, and DMSO-*d*_6_ was used for one of them, which was carefully collected for ^1^H NMR analyses. For all three experiments, the bottom sediment was washed with regular DMSO (2 mL × 3) and redissolved in water (5 mL) by using a volumetric flask. An aqueous solution (0.5 mL) was taken out and filtered through a 0.2 µM syringe filter; then, the obtained aqueous solution was charged for ion chromatography to determine cation centration.

## 4. Conclusions

In summary, we have introduced a new approach to extracting alkali metal cations by using previously reported sulfate-binding receptors. This was enabled by the characteristic sulfate binding properties of hexaurea receptors with high affinity and selectivity. Selective lithium extraction is dominated by a combination of various electrostatic interactions among lithium, sulfate, and the receptor. This is, to the best of our knowledge, the first example that a receptor bearing a single anion binding site can extract cations. Future work is ongoing to improve extraction selectivity and investigate the separation of other valuable cations by using tailor-made anion receptors.

## Figures and Tables

**Table 1:** Summary of the SLE efficiency of M_2_SO_4_ using various tripodal oligourea receptors ^a^.

	Li^+^	Na^+^	K^+^	Rb^+^	Cs^+^
^b^ Control	2%	2%	3%	1%	3%
**L^NO2^**	100%	62%	23%	17%	27%
**L^Me^**	100%	50%	18%	30%	64%
**L^C6^**	100%	48%	8%	15%	38%
^c^ **TL^C6^**	80%	33%	17%	15%	6%

^[a]^ SLE condition: 25 °C, 2 h, stirring rate: 1500 r/min, one equivalent of the receptor was used for hexaurea, and two equivalents of triurea were used. The data were determined based on IC. A solution of the receptor in DMSO (5 mM, 2 mL) and a solution of M_2_SO_4_ in water (625 mM, 16 μL) were used; the overall volume was 2.016 mL of DMSO with 0.8% water. The maximum concentration of M^+^ would be 4 mM in water (5 mL) if all solids were not dissolved. ^[b]^ Control indicates that only M_2_SO_4_ solids were used for SLE without adding any receptor. ^[c]^ A tripodal receptor consists of three urea units and terminal hexyl chains; the structure is shown in the Supplementary Materials (Scheme S1).

## Data Availability

The original contributions presented in the study are included in the article/supplementary material, further inquiries can be directed to the corresponding authors.

## Supplementary Materials

The following are available online at https://www.mdpi.com/article/10.3390/molecules29112445/s1, Schemes S1–S2: Synthetic procedure of making hexaurea and triurea receptors according to previously studies; Figures S1–S4: ^1^H NMR spectrum of **L^NO2^**, **L^Me^**, **L^C6^** and **TL^C6^**; Figures S5–S19: DFT-optimized structures and IGM plots of **L^C6^** with alkali metal sulfates. Tables S1–S4: Calculated binding distances for **L^C6^**·M_2_SO_4_. Table S5: Summary of control experiment for SLE studies; Tables S6–S17: Concentration of various cations remaining those are not dissolved after extraction by using receptors; Figures S20–S33: Stacked partial ^1^H NMR spectra of receptors and obtained DMSO solution with alkali metal salts after extraction; Figure S34: Stacked partial ^1^H NMR spectra after water wash; Figure S35: Stacked ^7^Li NMR spectra of Li_2_SO_4_ with one equivalent of **L^C6^** receptor by adding tetramethylammonium chloride; Figures S36–S40: Obtained HR-ESI-QTOF Mass spectrum for the complex of **L^C6^** with alkali metal sulfates.
