# Supplementary material for: Selective Solid–Liquid Extraction of Lithium Cation Using Tripodal Sulfate-Binding Receptors Driven by Electrostatic Interactions
Source: Molecules. 2024 May 22;29(11):2445. doi: 10.3390/molecules29112445 (PMC11173669; doi:10.3390/molecules29112445)
Supplement: Supplementary file 1 [file molecules-29-02445-s001.zip › molecules-3007370-supplementary.pdf]

Supporting Information (SI) for

Selective solid-liquid extraction of lithium cation using tripodal sulfate-binding receptors driven by electrostatic interactions

Ya-Zhi Chen[1], Ying-Chun He[1,2,*], Li Yan[3], Wei Zhao[1,*] and Biao Wu[1,*]

[1] *Key Laboratory of Medicinal Molecule Science and Pharmaceutics Engineering, Ministry of Industry and Information Technology, School of Chemistry and Chemical Engineering, Beijing Institute of Technology, Beijing 102488, China.*

Email: zhaochem@bit.edu.cn; wubiao@bit.edu.cn

[2] *Institute of Applied Chemistry, Shanxi University, Taiyuan 030006, China.*

E-mail: heyingchun@sxu.edu.cn

[3] *Analysis& Testing Center, Beijing Institute of Technology, Beijing102488, China*

Table of contents

S1. General Information

S2. Synthetic Procedures of Anion Binding Receptors

S3. Computational Studies

S4. Solid-Liquid Extraction Experiments

S5. Mass Spectrometry

S1. General Information

All starting materials and solvents were obtained from commercial sources (Beijing InnoChem, Aladdin, Macklin Science & Technology Co., Ltd.), which were used without further purification. ^{1}H spectra was recorded on Bruker AVANCE AV II-400/700 MHz spectrometer at 298 K. ^{1}H NMR chemical shifts were reported according to residual solvent peaks (^{1}H NMR: 2.50 ppm for DMSO-d_6;). The anion concentration in the aqueous solution was recorded on a Shine ion chromatography (CIC-D100, China). All aqueous solutions were prepared by using ultrapure water (18.25 MΩ·cm).

S2. Synthetic Procedures of Anion Binding Receptors

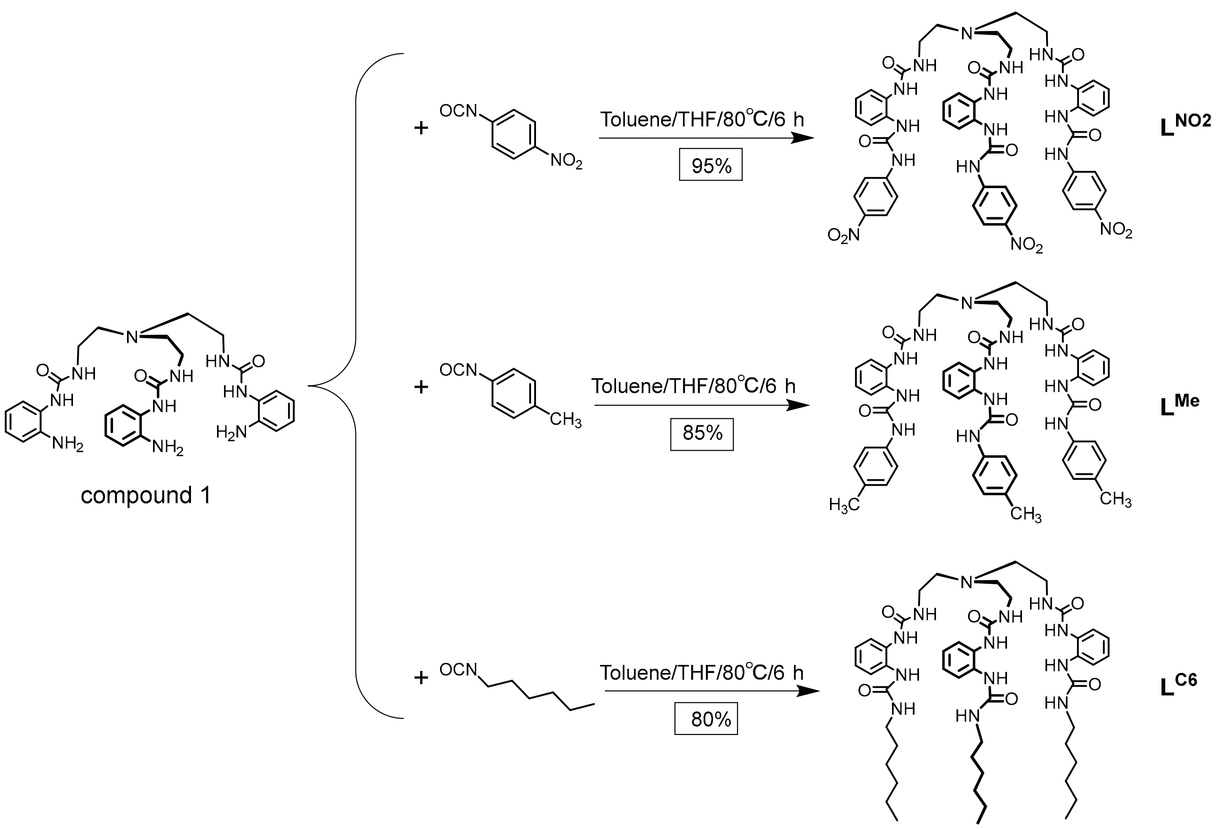

Scheme S1 Synthetic procedure of making hexaurea receptors according to previousl studies (*Angew. Chem., Int. Ed.,* **2011**, *50*, 486-490; *Inorg. Chem. Front.,* **2022**, *9*, 6091-6101).

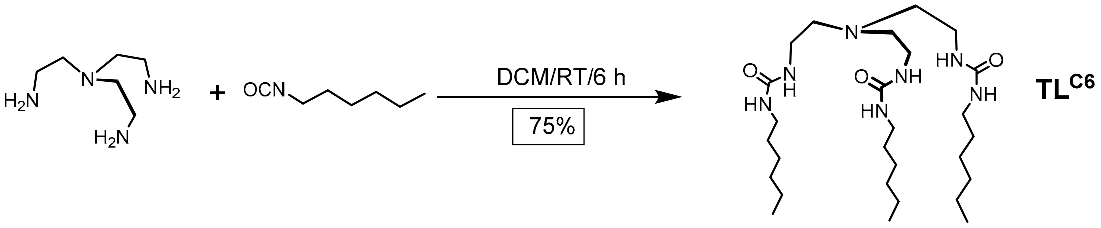

Scheme S2 Synthetic scheme of making triurea receptor.

Receptor L^{NO2}: Compound **1** (1.5 g, 2.7 mmol, 1.0 equiv.) was placed in a 500 mL double-necked, round-bottomed flask and suspended in THF (20 mL) and toluene (150 mL). The reaction mixture was heated to 80°C, and 4-nitrophenyl isocyanate (1.43 g, 8.7 mmol, 3.2 equiv.) in dry THF (15 mL) was added dropwise (2 drops per second). The mixture was stirred at the same temperature for 5 hours until compound **1** was consumed as monitored on thin layer chromatography (TLC, CH_2Cl_2:CH_3OH = 50:6). Then, the mixture was cooled down to room temperature, and precipitated solids was separated by filtration. The obtained yellow powder was further washed by THF and diethyl ether 3 times (30 mL). A pale yellow solid powder was obtained as receptor $\mathbf{L^{NO2}}$ (2.7 g), yield, 95%. The compound was characterized and confirmed by ^{1}H NMR according to previously reported data (*Angew. Chem., Int. Ed.,* **2011**, *50*, 486-490).

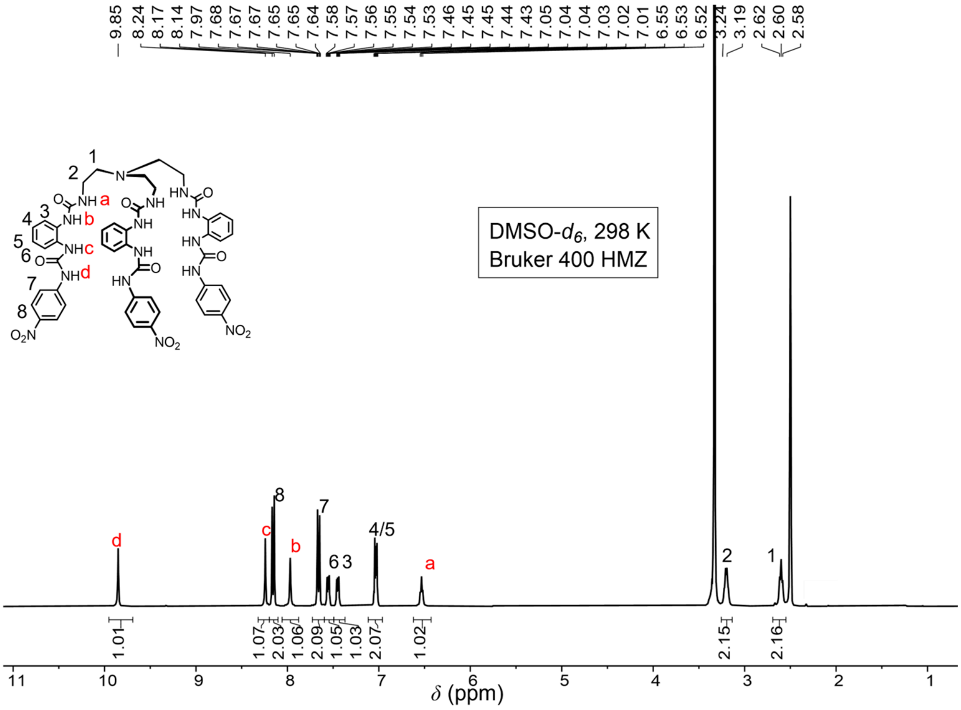

Figure S1 ^{1}H NMR spectrum (400 MHz, 298 K, DMSO-d_6) of $\mathbf{L^{NO2}}$.

Receptor L^{Me}: compound **1** (1.5 g, 2.7 mmol, 1.0 equiv.) was placed in a 500 mL double-necked, round-bottomed flask and suspend in dry THF (20 mL) and toluene (150 mL). The reaction was heated to 80 °C, and *P*-methylbenzene isocyanate (1.08 mL, 8.7 mmol, 3.2 equiv.) in dry THF (15 mL) was added dropwise over 30 min. The mixture was stirred at the same temperature for 6 hours until compound **1** was consumed based on TLC (CH_2Cl_2:CH_3OH = 50:6). The reaction mixture was cooled down to room temperature, and the precipitated solids was separated by filtration. The obtained yellow powder was further washed with THF and diethyl ether 3 times (30 mL). A white solid powder was yielded as receptor $\mathbf{L^{Me}}$ (2.2 g), yield, 85%. The compound was characterized and confirmed by ^{1}H NMR according to previously reported data (*Inorg. Chem. Front.*, **2022**, *9*, 6091-6101).

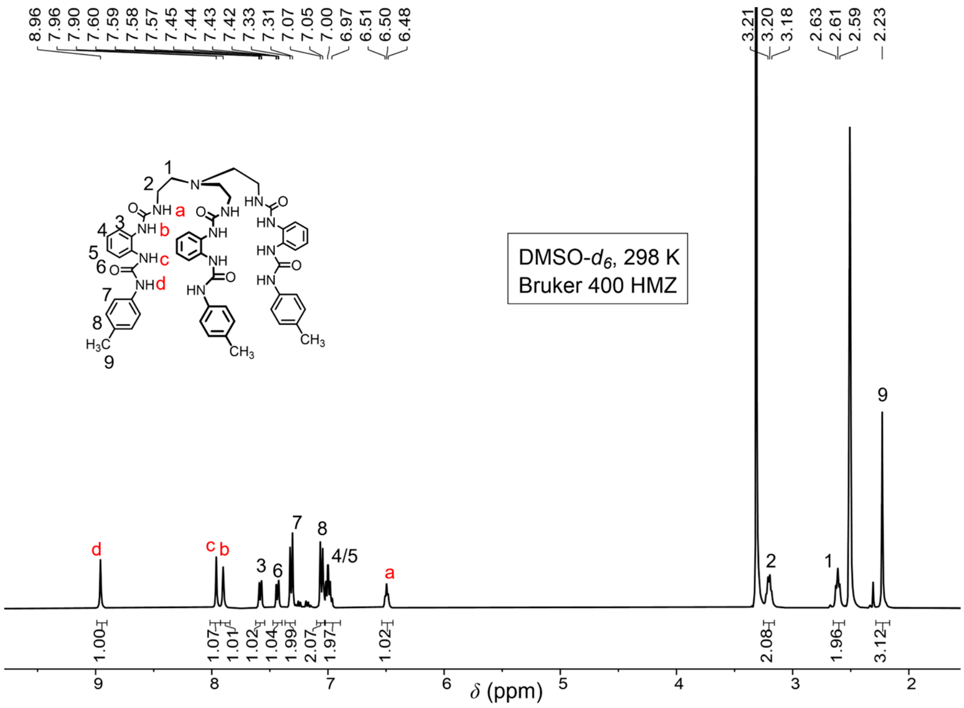

Figure S2 ^{1}H NMR spectrum (400 MHz, 298 K, DMSO-d_6) of $\mathbf{L^{Me}}$.

Receptor L^{C6}: compound **1** (1.5 g, 2.7 mmol, 1.0 equiv.) was placed in a 500 mL double-necked, round-bottomed flask and suspended in dry THF (20 mL) and toluene (150 mL). The reaction was heated to 80 °C, and hexylisocyanate (1.1 g, 8.7 mmol, 3.2 equiv.) in dry THF (15 mL) was added dropwise over 30 mins. The mixture was stirred at the same temperature for 6 hours until compound **1** was consumed based on TLC (CH_2Cl_2:CH_3OH = 50:3). The reaction mixture was cooled down to room temperature, and the precipitated solids were separated by filtration. The obtained off-white powder was further washed with THF and diethyl ether 3 times (30 mL). A

white solid powder was yielded as receptor **L^{C6}** (2.03 g), yield, 80%. The compound was characterized and confirmed by ^{1}H NMR according to previously reported data (*Inorg. Chem. Front.,* **2022**, *9*, 6091-6101).

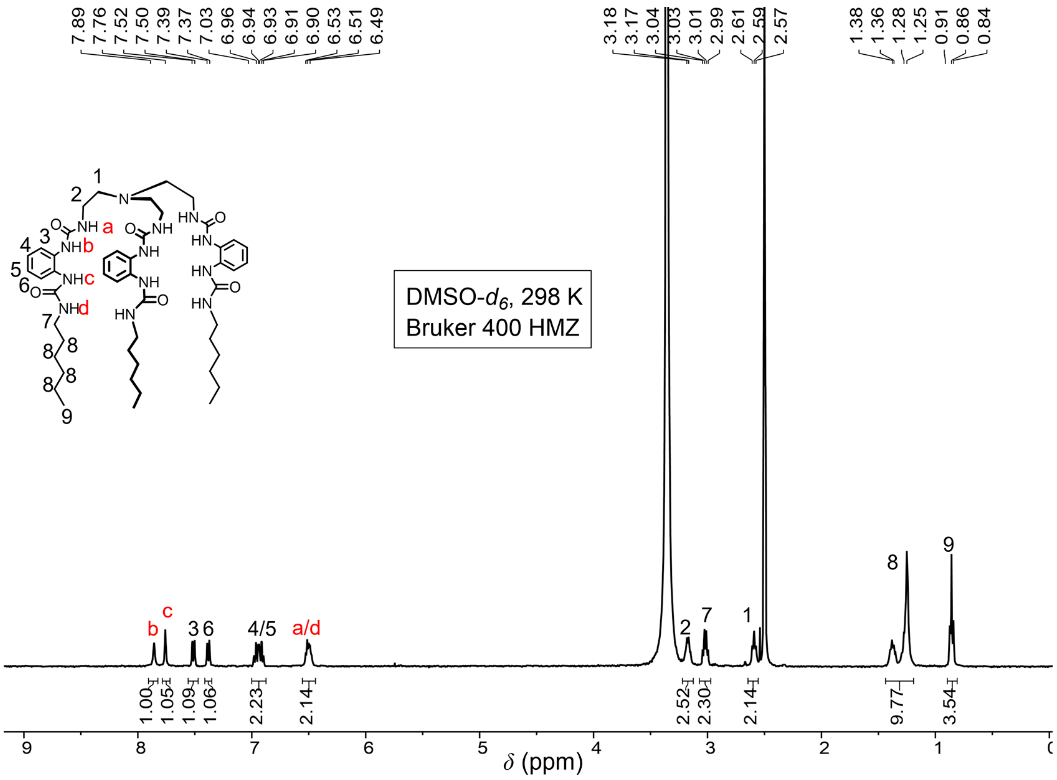

Figure S3 ^{1}H NMR spectrum (400 MHz, 298 K, DMSO-d_6) of **L^{C6}**.

Receptor TL^{C6}: tris(2-aminoethyl)amine (tren, 1.1 mL, 7.4 mmol, 1 equiv.) was placed in a 250 mL round-bottomed flask and dissolved in DCM (45 mL), A solution of hexylisocyanate (3 g, 23.6 mmol, 3.2 equiv.) in dry DCM (30 mL) was added into the flask by using a dropping funnel (2 seconds per droplet). The mixture was stirred for 6 h at room temperature and monitored by TLC(CH_2Cl_2:CH_3OH = 50:3). The gel-like precipitates were separated by filtration and washed with DCM and diethyl ether 3 times (30 mL). The obtained off-white powder was further dried over vacuum dried and isolated as receptor **TL^{C6}** (2.9 g). Yield, 75 %. The compound was characterized and confirmed by ^{1}H NMR according to previously reported data (*Chem. Commun.*, **2013**, *49*, 9119-9121).

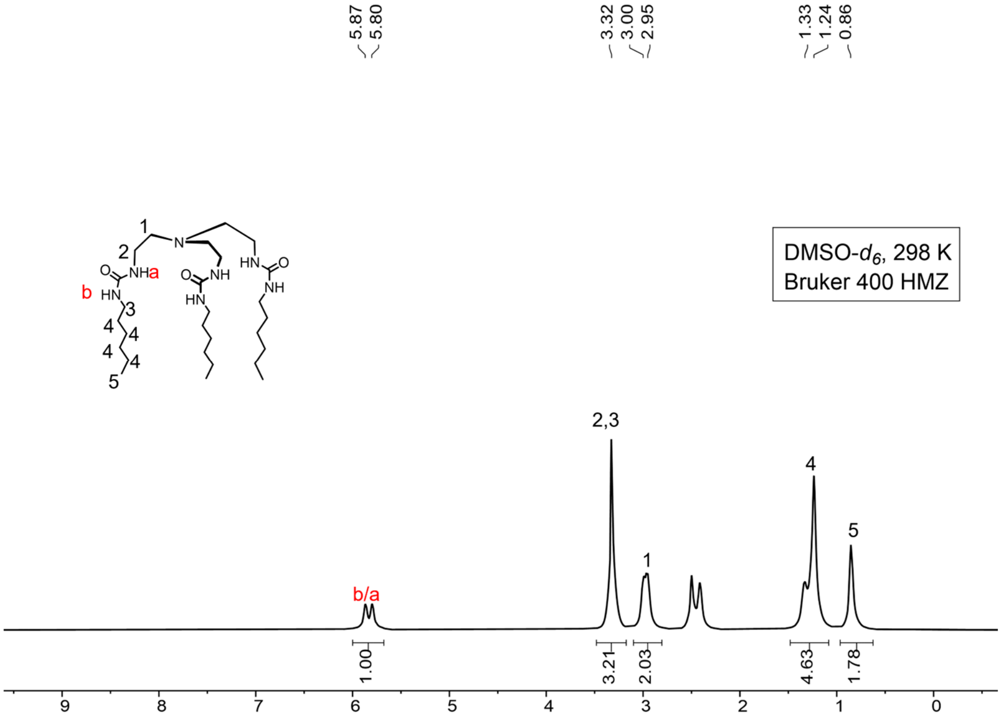

Figure S4 ^{1}H NMR spectrum (400 MHz, 298 K, DMSO-d_6) of $\mathbf{TL^{C6}}$.

S3. Computational Studies

DFT calculations were perfomed for the structural optimization of the complexation of $\mathbf{L^{C6}}$ with various M_2SO_4 salts using Spartan 20 at the theory level of B3LYP/6-31G(D) in DMSO continuum with the Conductor-like Polarizable Continuum Model. The sulfate binding geometry of $\mathbf{L^{C6}}$ receptor with tetramethylammonium (TMA) countercations was firstly calculated, in which the obtained structure (including hydrogen bonding interactions and relative location of TMA cations) is comparable to the previously reported crystal structure (*Angew. Chem., Int. Ed.,* **2011**, *50*, 486-490), suggesting that the used method for DFT calculations is reliable and subsequently applied for the calculation with M_2SO_4 salts.

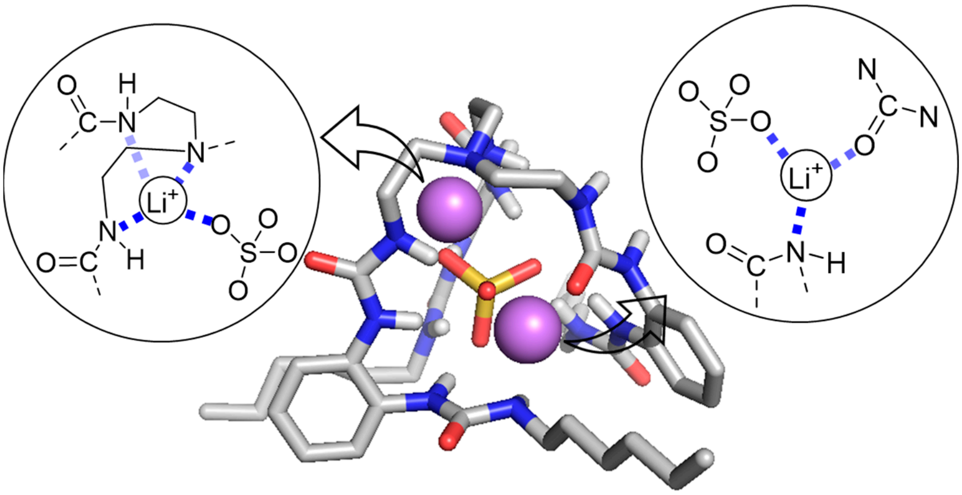

Figure S5 DFT-optimized structure for the Li_2SO_4 binding complex by using Spartan 20 at the theory level of B3LYP/6-31G(D).

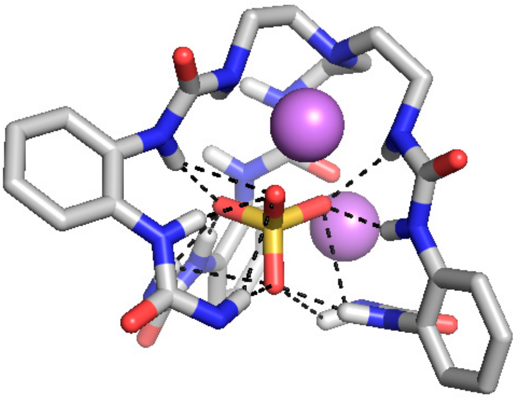

Figure S6 Hydrogen bonding networks seen in the calculated Li_2SO_4 binding complex where eight N-H···O hydrogen bonds are shown.

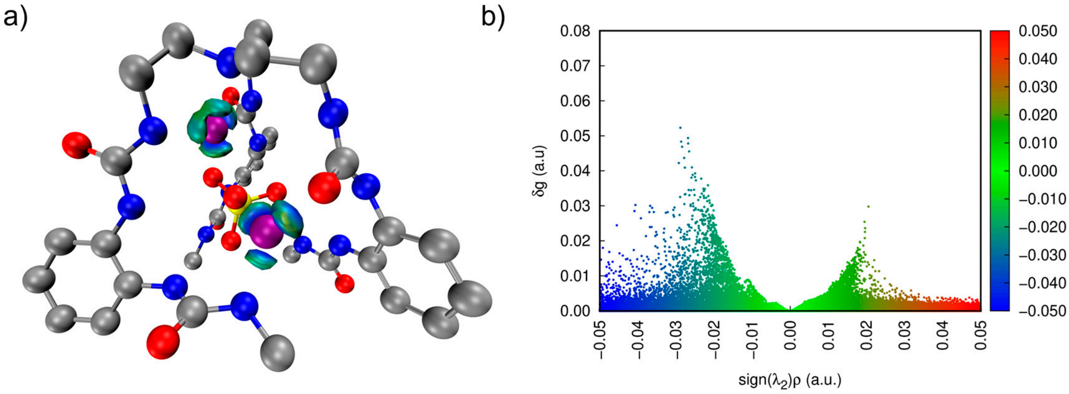

Figure S7 (a) IGM isosurface and (b) IGM scatterplot illustrating the interaction of sulfate, carbonyl oxygen and lithium cations. The van der Waals force and electrostatic force are dominant. Color coding in the range of $-0.5 < \rho$ sign(λ_2) < 0.5 a.u., atom colors: grey = C, blue = N, red = O, yellow = S, purple = Li.

Table *S1* Calculated Binding Distances for $L^{C6}\cdot Li_2SO_4$.

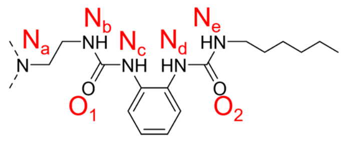

L ^{C6}

Ion pair	Atoms	Distance (Å)
	$N_a\cdots Li$	2.23
	$N_{b1}\cdots Li$	2.23
	$N_{b2}\cdots Li$	2.26
	$N_c\cdots Li$	-
Li_2SO_4	$N_e\cdots Li$	2.16
	$O_1\cdots Li$	1.88
	$O_1(SO_4^{2-})\cdots Li$	1.88
	$O_2(SO_4^{2-})\cdots Li$	1.97
	$O_3(SO_4^{2-})\cdots Li$	-
	Average(Å)	2.09

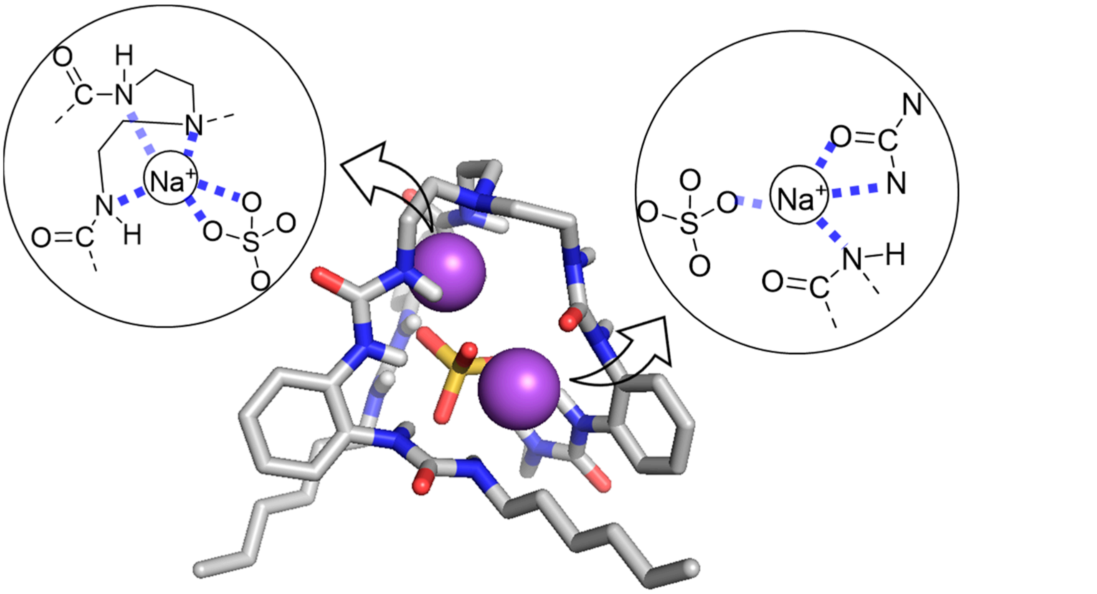

Figure S8 DFT-optimized structure for the Na_2SO_4 binding complex by using Spartan 20 at the theory level of B3LYP/6-31G(D).

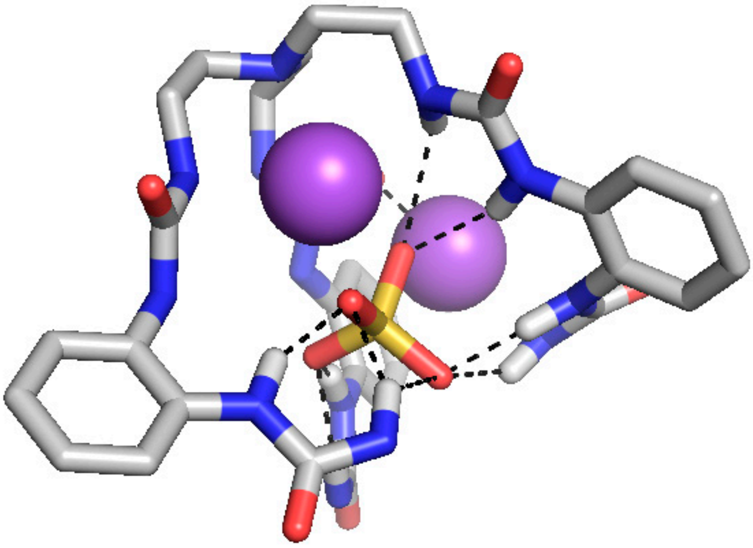

Figure S9 Hydrogen bonding networks seen in the calculated Na_2SO_4 binding complex where eight N-H···O hydrogen bonds are shown.

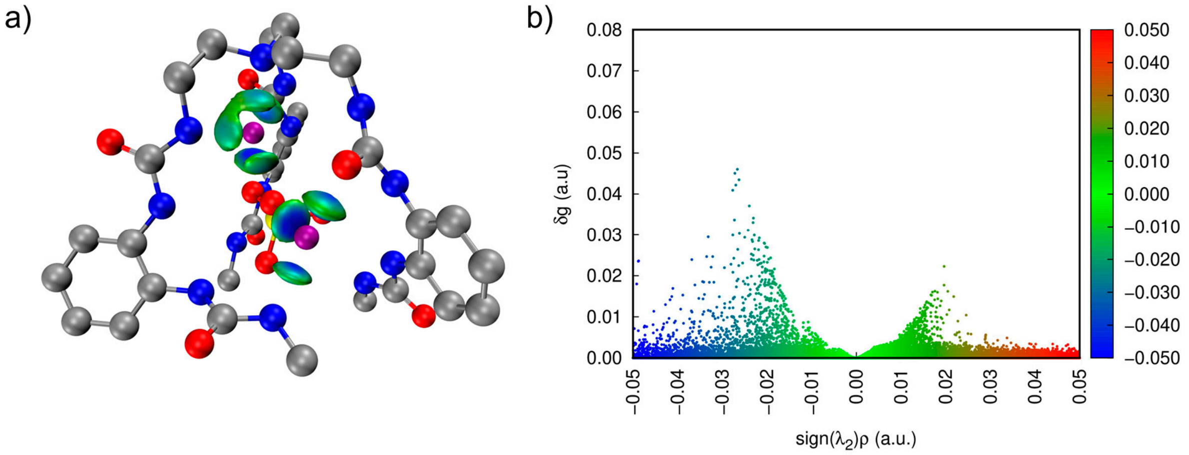

Figure S10 (a) IGM isosurface and (b) IGM scatterplot illustrating the interaction of sulfate, carbonyl oxygen and sodium cations. The van der Waals force and electrostatic force are dominant. Color coding in the range of $-0.5 < \rho \ \mathrm{sign}(\lambda_2) < 0.5$ a.u., atom colors: grey = C, blue = N, red = O, yellow = S, purple = Na.

Table *S2* Calculated Binding Distances for $L^{C6}\cdot Na_2SO_4$.

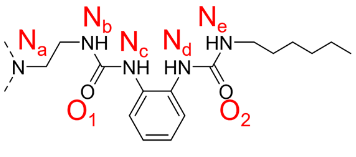

L C6

Ion pair	Atoms	Distance (Å)
	$N_a\cdots Na$	2.48
	$N_{b1}\cdots Na$	2.51
	$N_c\cdots Na$	-
	$N_e\cdots Na$	2.51
Na_2SO_4	$O_1\cdots Na$	2.28
	$O_1(SO_4^{2-})\cdots Na$	2.25
	$O_2(SO_4^{2-})\cdots Na$	2.34
	$O_3(SO_4^{2-})\cdots Na$	2.40
	Average(Å)	2.40

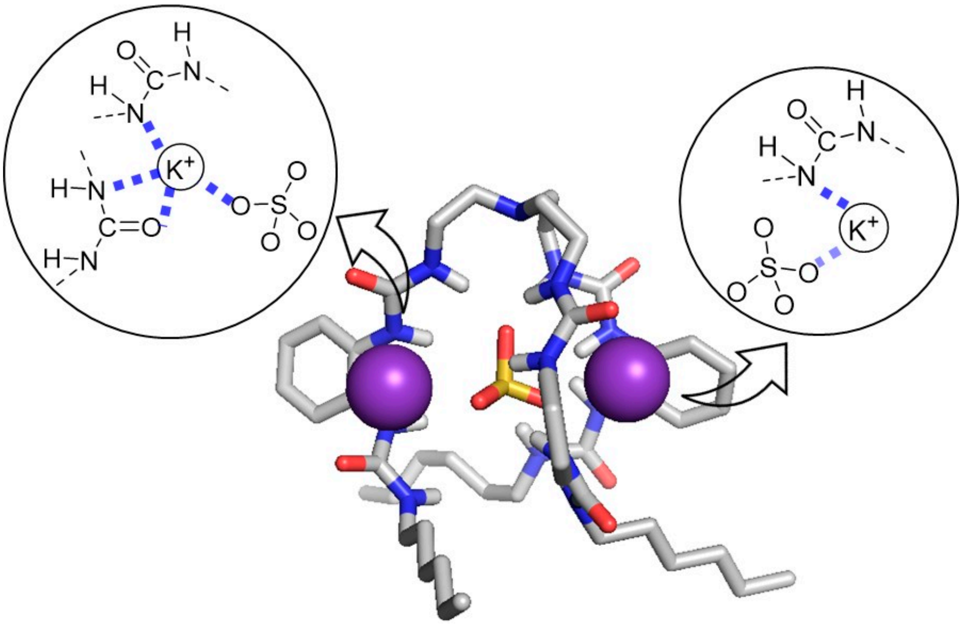

Figure S11 DFT-optimized structure for the K_2SO_4 binding complex by using Spartan 20 at the theory level of B3LYP/6-31G(D), where two K^+ cations are seen to reside outside the cavity.

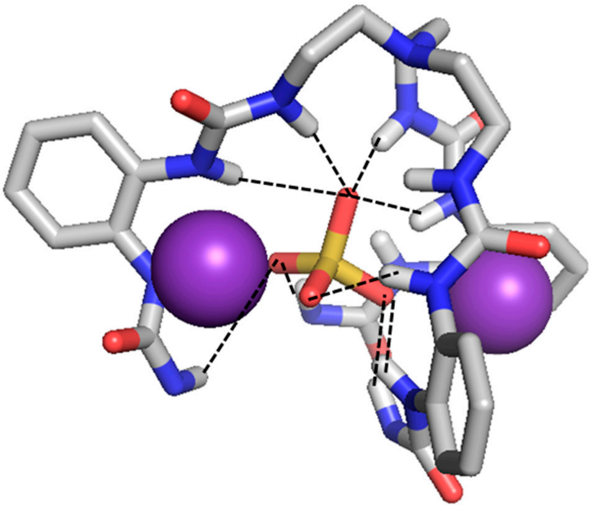

Figure S12 Hydrogen bonding networks seen in the calculated Na_2SO_4 binding complex where eleven N-H···O hydrogen bonds are shown.

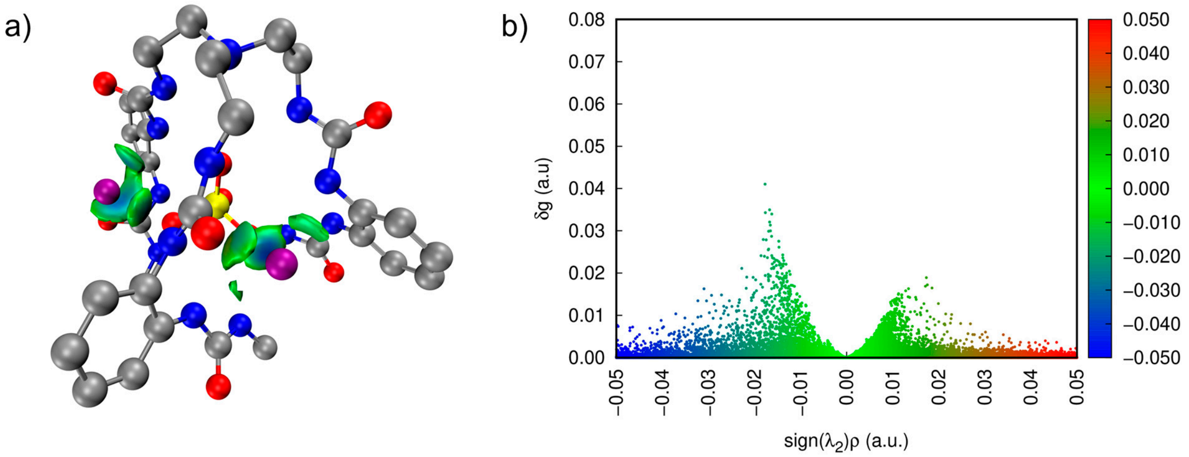

Figure S13 (a) IGM isosurface and (b) IGM scatterplot illustrating the interaction of sulfate, carbonyl oxygen and potassium cations. The van der Waals force are dominant. Color coding in the range of -0.5 < ρ sign(λ_2) < 0.5 a.u., atom colors: grey = C, blue = N, red = O, yellow = S, purple = K.

Table *S3* Calculated Binding Distances for $L^{C6} \cdot K_2SO_4$.

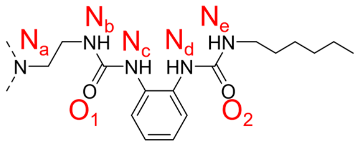

L C6

Ion pair	Atoms	Distance (Å)
	$N_c \cdots K$	2.90
	$N_d \cdots K$	2.97
	$N_e \cdots K$	-
	$O_1 \cdots K$	-
K_2SO_4	$O_{11} \cdots K$	-
	$O2 \cdots K$	2.79
	$O_1(SO_4^{2-}) \cdots K$	2.62
	$O_2(SO_4^{2-}) \cdots K$	2.64
	Average(Å)	2.78

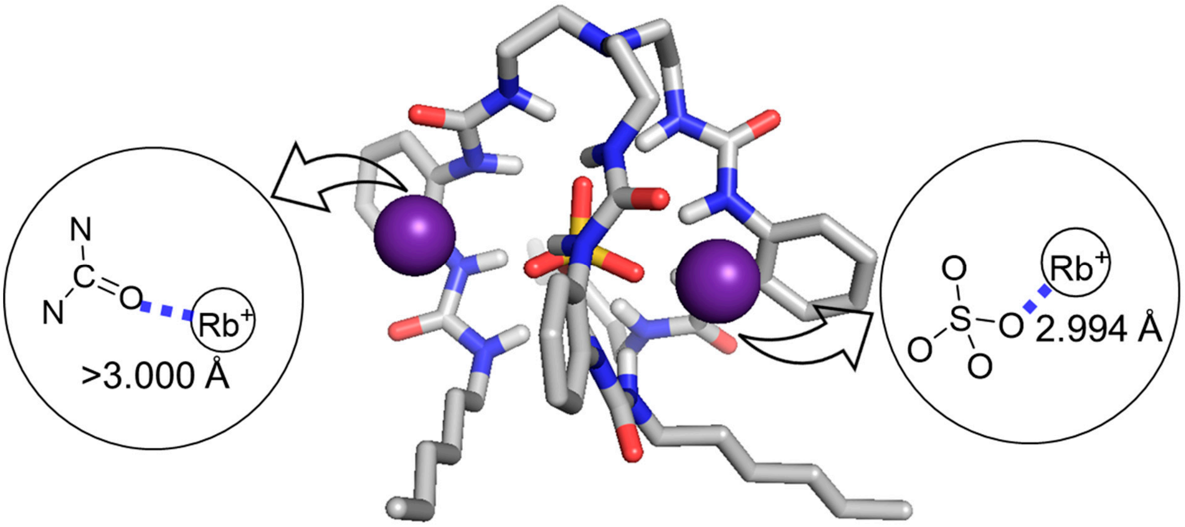

Figure S14 DFT-optimized structure for the Rb_2SO_4 binding complex by using Spartan 20 at the theory level of B3LYP/6-31G(D), where two Rb^+ cations are seen to reside outside the cavity.

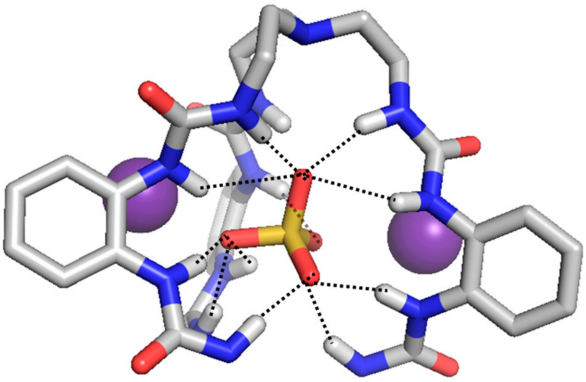

Figure S15 Hydrogen bonding networks seen in the calculated Rb_2SO_4 binding complex where eleven N-H···O hydrogen bonds are shown.

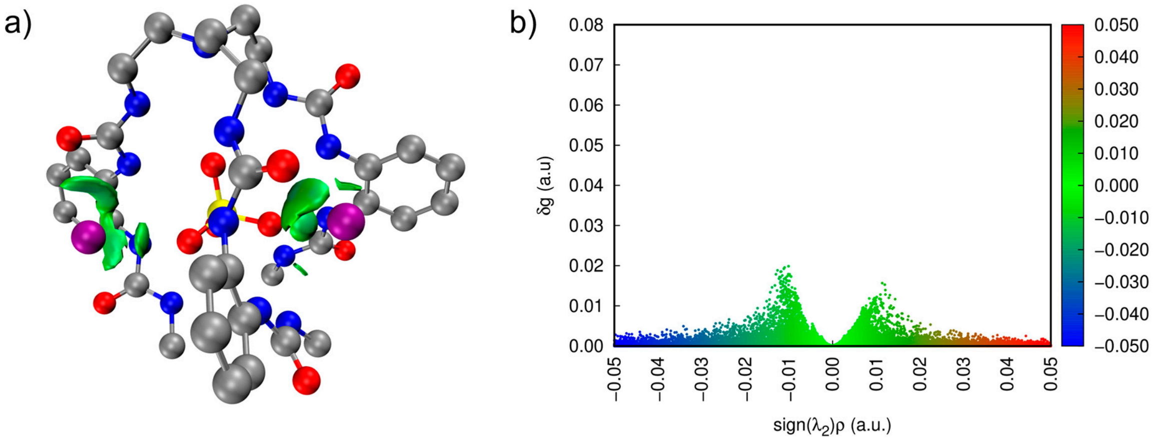

Figure S16 (a) IGM isosurface and (b) IGM scatterplot illustrating the interaction of sulfate, carbonyl oxygen and Rb^+ cations. Weak van der Waals force are seen. Color coding in the range of $-0.5 < \rho$ sign(λ_2) < 0.5 a.u., atom colors: grey = C, blue = N, red = O, yellow = S, purple = Rb.

Table *S4* Calculated Binding Distances and Numbers for $L^{C6}\cdot Rb_2SO_4$.

Ion pair	Atoms	Distance(Å)
	$O_1(SO_4^{2-})\cdots Rb$	2.98
Rb_2SO_4	$O_2(SO_4^{2-})\cdots Rb$	-
	Average(Å)	2.98

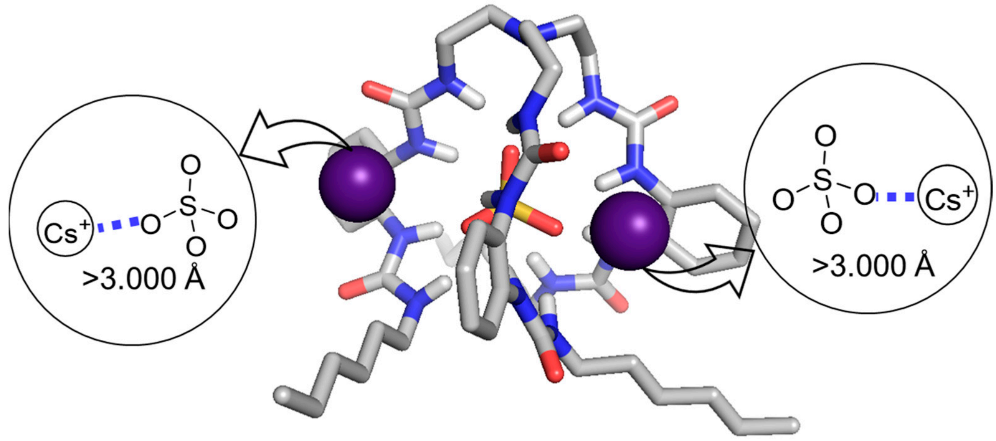

Figure S17 DFT-optimized structure for the Cs_2SO_4 binding complex by using Spartan 20 at the theory level of B3LYP/6-31G(D), where two Cs^+ cations are seen to reside outside the cavity.

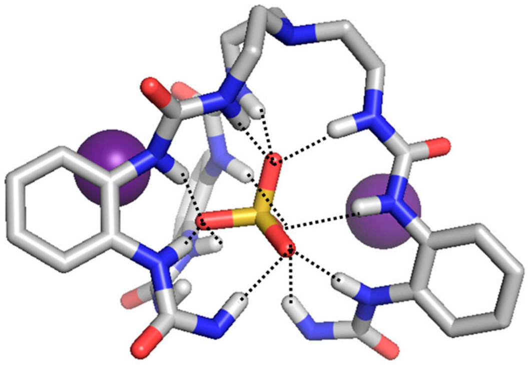

Figure S18 Hydrogen bonding networks seen in the calculated Cs_2SO_4 binding complex where twelve N-H···O hydrogen bonds are shown.

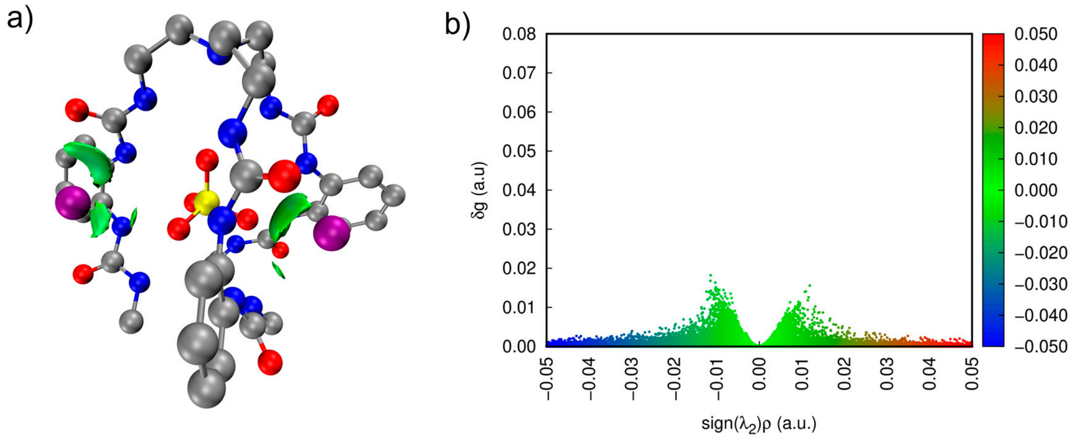

Figure S19 (a) IGM isosurface and (b) IGM scatterplot illustrating the interaction of sulfate, carbonyl oxygen and Rb^+ cations. Negligible van der Waals force is seen. Color coding in the range of $-0.5 < \rho$ sign(λ_2) < 0.5 a.u., atom colors: grey = C, blue = N, red = O, yellow = S, purple = Cs.

S4. Solid-Liquid Extraction Experiments

S4.1 General solid-liquid extraction procedures

General solid-liquid extraction procedure: A solution of the receptor (5 mM, 2 mL, DMSO) is exposed to an aqueous solution (16 μL) containing targeted alkali metal cations (e.g., Li_2SO_4, Na_2SO_4, K_2SO_4, Rb_2SO_4, Cs_2SO_4, 625 mM). The added M_2SO_4 solids are seen to rapidly precipitated. The prepared solution mixture is stirred at 25ºC for 2 hours with a stirring rate of 1500 r/min. The solution and undissolved solids are separated upon centrifugation. The organic layer is carefully collected and redissolved in DMSO-d_6 for ^{1}H NMR analyses. The bottom sediment is washed with regular DMSO (2 mL × 3) and redissolved in water (5 mL) by using a volumetric flask. An aqueous solution (0.5 mL) is taken and subjected to a 0.2 μM syringe filter, then the obtained aqueous solution is charged for ion chromatography to determine cation centration.

A solution of receptor in DMSO (5 mM, 2 mL) and a solution of M_2SO_4 in water (625 mM, 16 μL) were used, the overall volume is 2.016 mL of DMSO with 0.8% water. The maximum concentration of M^+ would be 4 mM in water (5 mL) if all the solids were not dissolved.

Table S5 Summary of control experiment for SLE studies, i.e., remaining concentration of alkali metal cation extraction without using any receptor. Condition: 5.0 hours, 25°C, stirring rate: 1500 r/min, in pure DMSO. M_2SO_4 was added as solids without dissolving in water.

	Initial concentration (mM)	After extraction (mM)
Li^+	4.03±0.06	4.01±0.02
Na^+	4.12±0.05	4.06±0.04
K^+	4.08±0.02	4.03±0.03
Rb^+	3.98±0.05	3.91±0.07
Cs^+	4.06±0.07	3.99±0.05

Table S6 Concentration of various cations remaining those are not dissolved after extraction by using $\mathbf{L}^{\mathbf{NO2}}$. SLE condition, 5.0 hours, 25°C, stirring rate: 1500 r/min, pure DMSO. M_2SO_4 was added as solids without dissolving in water.

Cation	After extraction (mM)	Extraction efficiency
Li^+	3.98±0.03	0%
Na^+	4.07±0.06	0%
K^+	4.02±0.06	0%
Rb^+	3.95±0.02	0%
Cs^+	4.00±0.05	0%

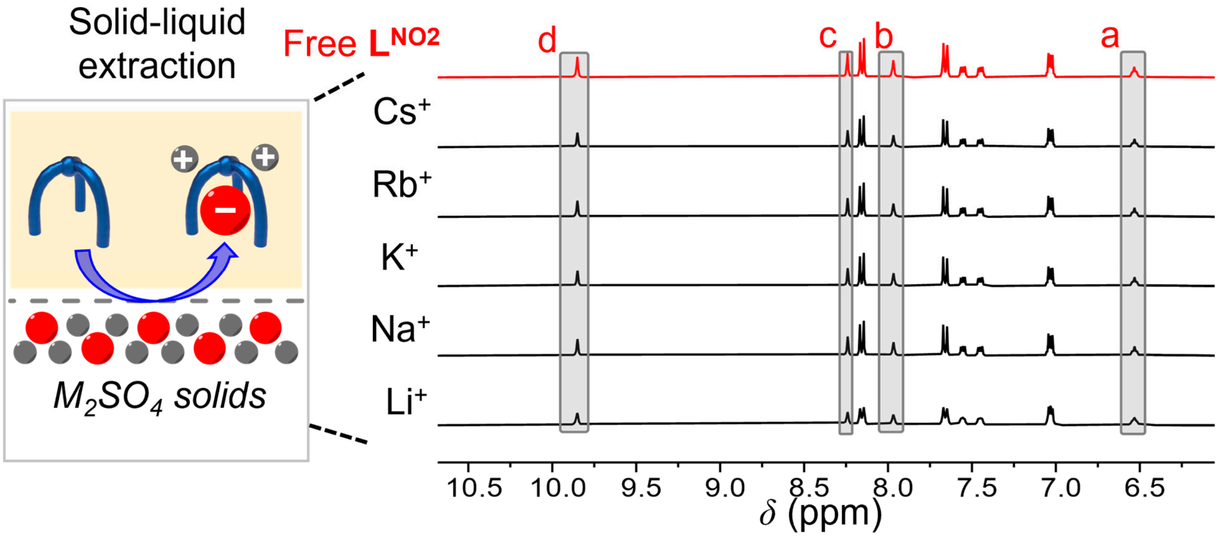

Figure S20 Stacked partial ^{1}H NMR spectra (400 MHz, DMSO-d_6, 298 K), from top to bottom: receptor L^{NO2} and obtained DMSO solution after extraction in the presence of one equivalent of different alkali sulfate solids (SLE condition, 5.0 hours, 25°C, stirring rate: 1500 r/min, pure DMSO. M_2SO_4 was added as solids without dissolving in water). Based on the ^{1}H NMR spectra, , it reveals that the receptor cannot extract alkali sulfate solids without adding trace amount of water.

Table S7 Concentrations of alkali remaining after SLE by adding alkali sulfate solids only. Condition: 5.0 hours, 25°C, stirring rate: 1500 r/min, in DMSO with 0.8% water.

Cation	Initial concentration(mM)	After extraction(mM)
Li^+	4.03±0.06	3.95±0.04
Na^+	4.12±0.05	4.05±0.07
K^+	4.08±0.02	3.97±0.11
Rb^+	3.98±0.05	3.94±0.03
Cs^+	4.06±0.07	3.96±0.04

Table S8 Concentrations of alkali cations remaining after extraction by using L^{NO2} with and without two equivalents of 18-crown-6 ether macrocycles. SLE condition: 5.0 hours, 25°C, stirring rate, 1500 r/min, in DMSO with 0.8% water.

Cation	After extraction (mM) (18C6+M_2SO_4)	Extraction efficiency (18C6+M_2SO_4)	After extraction (mM) (M_2SO_4)	Extraction efficiency (M_2SO_4)
Li^+	ND	100%	ND	100%
Na^+	1.03±0.06	75%	0.98±0.12	76%
K^+	2.77±0.04	32%	2.76±0.03	32%
Rb^+	1.67±0.01	42%	1.91±0.05	52%
Cs^+	1.21±0.14	70%	1.78±0.03	56%

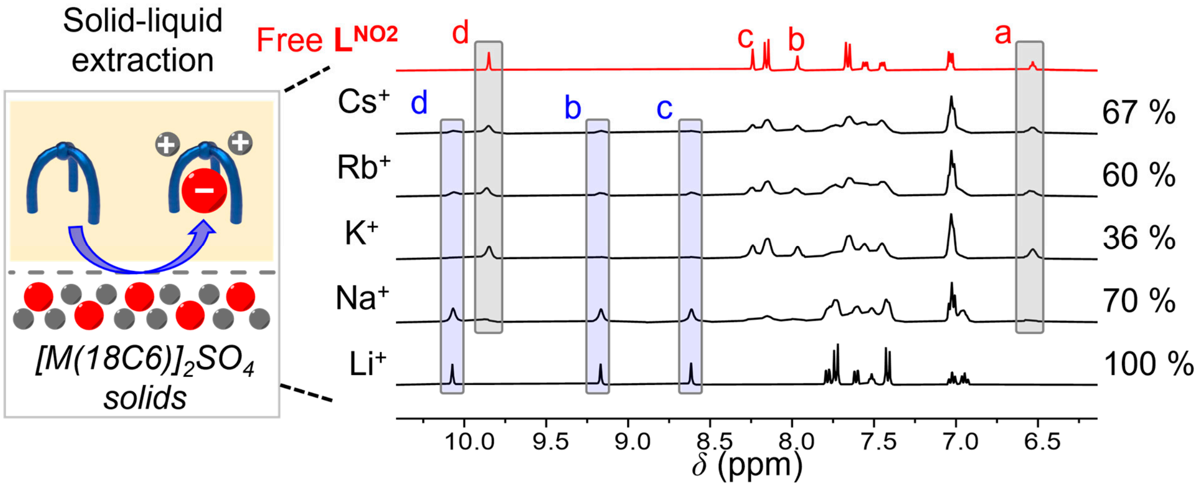

Figure S21 Stacked partial ^{1}H NMR spectra (400 MHz, DMSO-*d*6, 298 K) of receptor L^{NO2} and obtained DMSO solution after extraction in the presence of two equivalents of 18-crown-6 ether macrocycles. SLE condition: 5.0 hours, 25°C, stirring rate, 1500 r/min, in DMSO with 0.8% water. The peaks within grey frameworks are assiged to free receptor, and the peaks within light purple frameworks are assigned to complexed receptors after extraction. The determined extraction efficiency are shown on the right as determined based on NMR spectra.

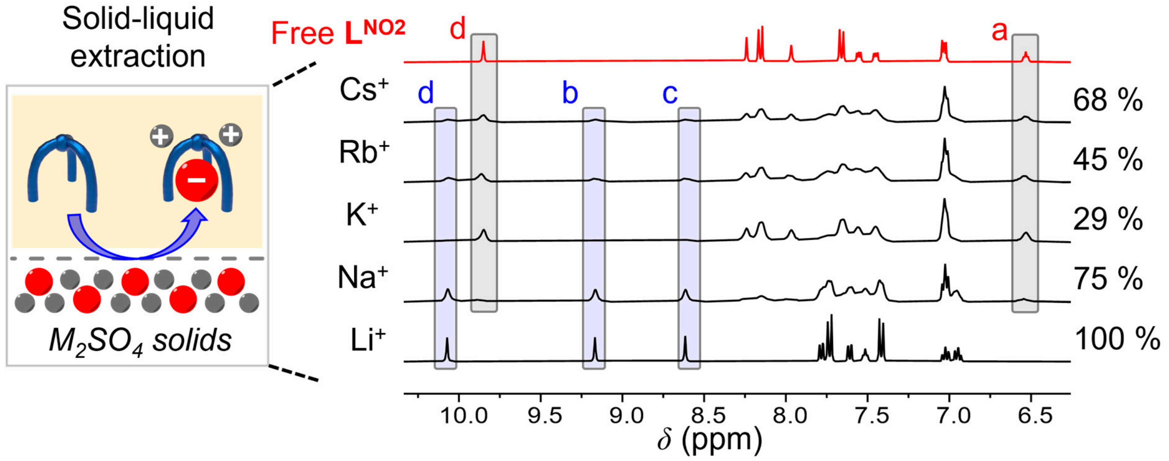

Figure S22 Stacked partial ^{1}H NMR spectra (400 MHz, DMSO-*d*6, 298 K) of receptor L^{NO2} and obtained DMSO solution after extraction without adding 18-crown-6 ether macrocycle. SLE condition: 5.0 hours, 25°C, stirring rate, 1500 r/min, in DMSO with 0.8% water. The peaks within grey frameworks are assiged to free receptor, and the peaks within light purple frameworks are assigned to complexed receptors after extraction. The determined extraction efficiency are shown on the right as determined based on NMR spectra.

Table S9 Concentrations of alkali cations remaining after extraction by using $\mathbf{L^{NO2}}$. SLE condition: 5.0 hours, 50°C, stirring rate, 1500 r/min, in DMSO with 0.8% water.

Cation	After extraction (mM)	Extraction efficiency
Li^+	ND	100%
Na^+	0.74±0.09	82%
K^+	2.16±0.11	47%
Rb^+	2.58±0.05	35%
Cs^+	1.42±0.07	65%

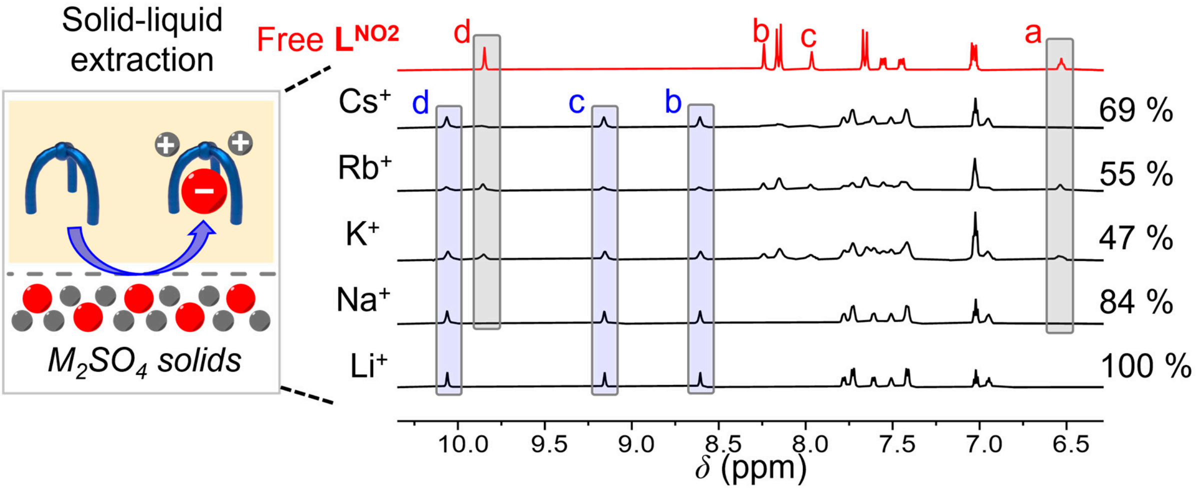

Figure S23 Stacked partial ^{1}H NMR spectra (400 MHz, DMSO-d_6, 298 K) of receptor L^{NO2} and obtained DMSO solution after extraction. SLE condition: 5.0 hours, 50°C, stirring rate, 1500 r/min, in DMSO with 0.8% water. The peaks within grey frameworks are assiged to free receptor, and the peaks within light purple frameworks are assigned to complexed receptors after extraction. The determined extraction efficiency are shown on the right as determined based on NMR spectra. The overall extraction selectivity for lithium cation is decreased as increasing temperature.

Table S10 Concentrations of alkali cations remaining after extraction by using **L^{NO2}** upon changing SLE time. SLE condition: 25°C, stirring rate, 1500 r/min, in DMSO with 0.8% water.

Time (h)	After extraction (Li_2SO_4) (mM)	Extraction efficiency (Li_2SO_4)	After extraction (Na_2SO_4) (mM)	Extraction efficiency (Na_2SO_4)
0.5 h	1.59±0.08	60%	2.23±0.05	46%
1.0 h	1.08±0.05	73%	1.51±0.11	64%
1.5 h	0.40±0.04	90%	1.62±0.12	60%
2.0 h	0.06±0.03	99%	1.55±0.05	62%
3.0 h	0.01±0.01	100%	1.19±0.02	72%
5.0 h	0.03±0.03	100%	1.15±0.10	72%

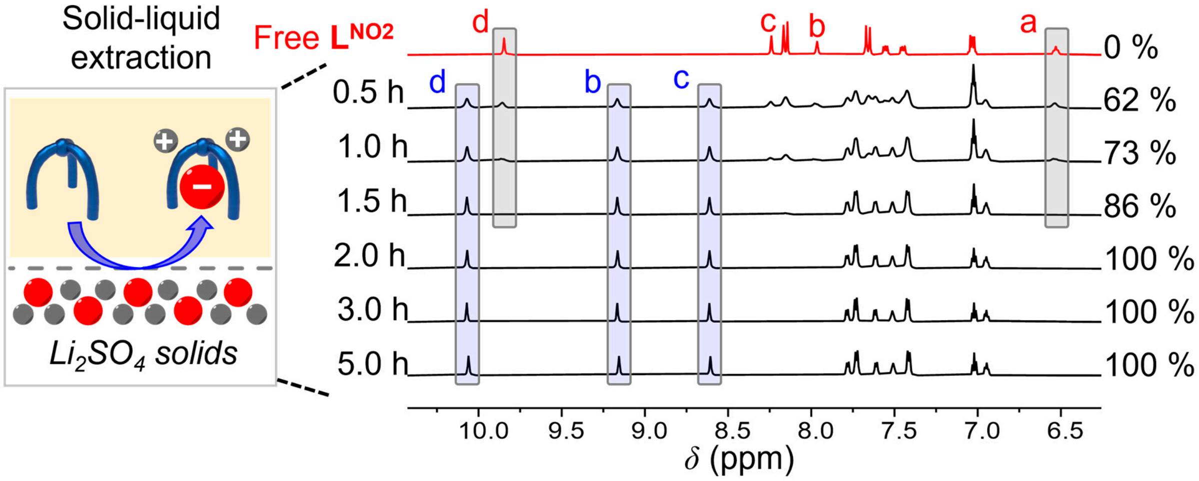

Figure S24 Stacked partial ^{1}H NMR spectra (400 MHz, DMSO-d_6, 298 K) of receptor L^{NO2} and obtained DMSO solution after extraction with Li_2SO_4 salts. SLE condition: 25°C, stirring rate, 1500 r/min, in DMSO with 0.8% water. The peaks within grey frameworks are assiged to free receptor, and the peaks within light purple frameworks are assigned to complexed receptors after extraction. The determined extraction efficiency are shown on the right as determined based on NMR spectra.

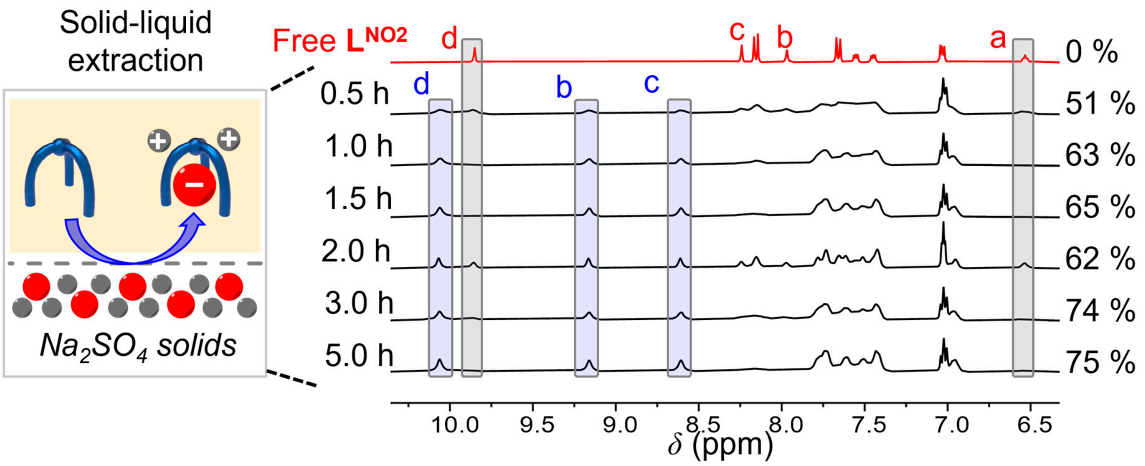

Figure S25 Stacked partial ^{1}H NMR spectra (400 MHz, DMSO-d_6, 298 K) of receptor L^{NO2} and obtained DMSO solution after extraction with Na_2SO_4 salts. SLE condition: 25°C, stirring rate, 1500 r/min, in DMSO with 0.8% water. The peaks within grey frameworks are assiged to free receptor, and the peaks within light purple frameworks are assigned to complexed receptors after extraction. The determined extraction efficiency are shown on the right as determined based on NMR spectra.

Table S11 Concentrations of alkali cations remaining after extraction by using $\mathbf{L^{NO2}}$ upon changing stirring rate. SLE condition: 25°C, stirring rate, 1.0 hour, in DMSO with 0.8% water.

Stir rate r/min	After extraction (Li_2SO_4) (mM)	Extraction efficiency (Li_2SO_4)	After extraction (Na_2SO_4) (mM)	Extraction efficiency (Na_2SO_4)
500	1.59±0.08	65%	1.73±0.05	58%
1000	1.08±0.05	70%	1.65±0.03	60%
1500	1.08±0.05	73%	1.51±0.11	64%

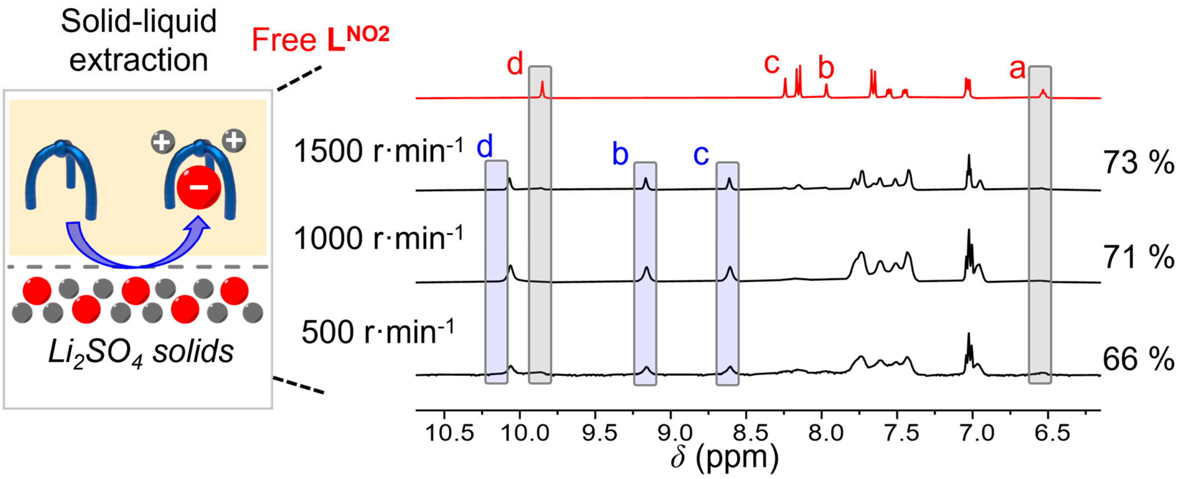

Figure S26 Stacked partial ^{1}H NMR spectra (400 MHz, DMSO-*d*6, 298 K) of receptor L^{NO2} and obtained DMSO solution after extraction with Li_2SO_4 salts by chaing the stirring rate SLE condition: 25°C, in DMSO with 0.8% water. The peaks within grey frameworks are assiged to free receptor, and the peaks within light purple frameworks are assigned to complexed receptors after extraction. The determined extraction efficiency are shown on the right as determined based on NMR spectra.

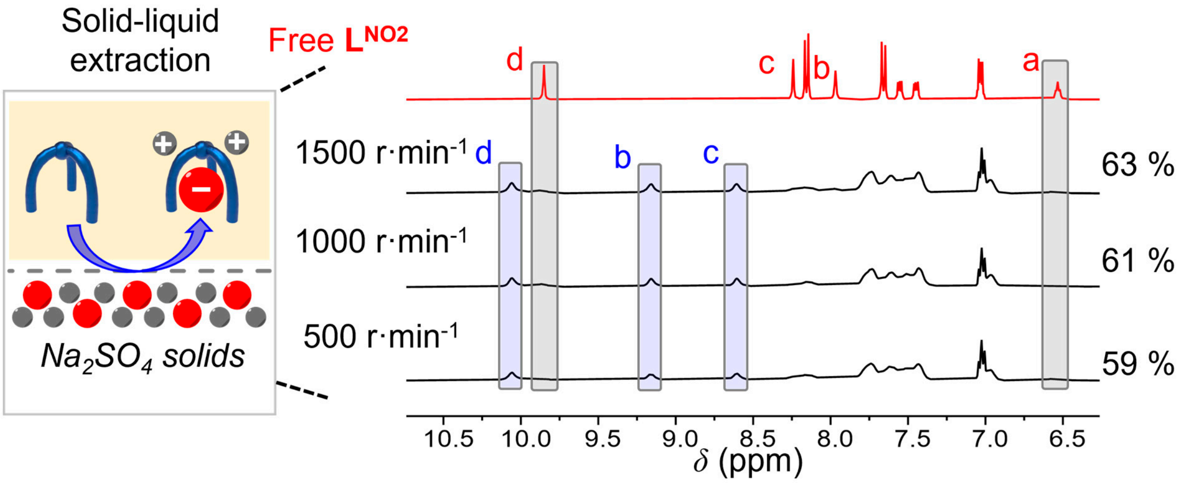

Figure S27 Stacked partial ^{1}H NMR spectra (400 MHz, DMSO-*d*6, 298 K) of receptor L^{NO2} and obtained DMSO solution after extraction with Na_2SO_4 salts by chaing the stirring rate SLE condition: 25°C, in DMSO with 0.8% water. The peaks within grey frameworks are assiged to free receptor, and the peaks within light purple frameworks are assigned to complexed receptors after extraction. The determined extraction efficiency are shown on the right as determined based on NMR spectra.

Table S12 Concentrations of alkali cations remaining after extraction by using $\mathbf{L^{NO2}}$. SLE condition: 2 hours, 25°C, stirring rate, 1500 r/min, in DMSO with 0.8% water.

Cation	After extraction (mM)	Extraction efficiency
Li^+	None	100%
Na^+	1.60±0.06	61%
K^+	3.06±0.09	25%
Rb^+	3.29±0.03	17%
Cs^+	2.84±0.05	30%

Table S13 Concentrations of alkali cations remaining after extraction by using $\mathbf{L^{Me}}$ and $\mathbf{L^{C6}}$. SLE condition: 2 hours, 25°C, stirring rate, 1500 r/min, in DMSO with 0.8% water.

Cation	After extraction (mM) (L^{Me})	Extraction efficiency (L^{Me})	After extraction (mM) (L^{C6})	Extraction efficiency (L^{C6})
Li^+	None	100%	None	100%
Na^+	2.30±0.04	44%	2.26±0.09	45%
K^+	3.30±0.03	19%	3.67±0.04	10%
Rb^+	2.82±0.01	29%	3.38±0.05	15%
Cs^+	1.54±0.02	62%	2.59±0.11	36%

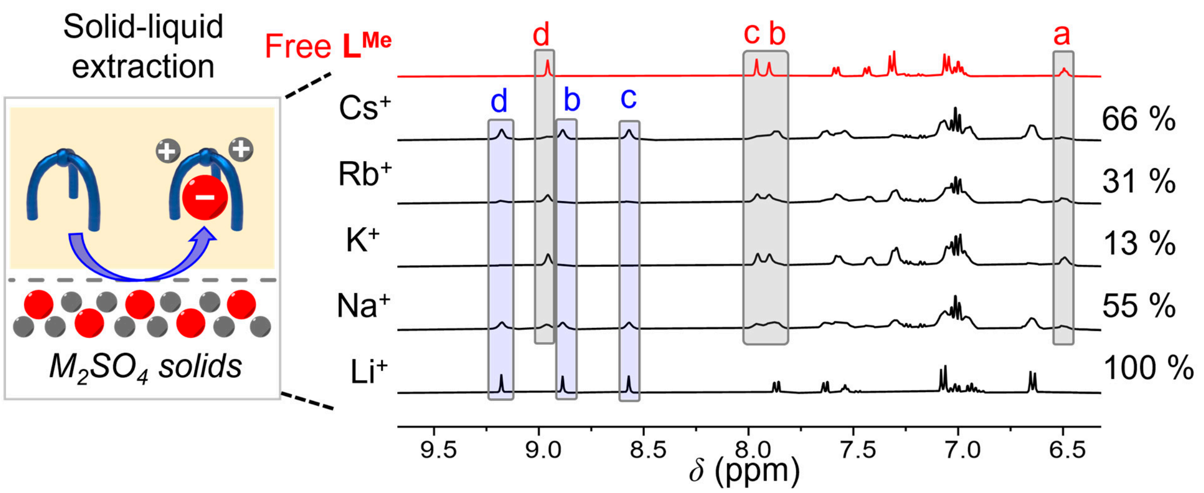

Figure S28 Stacked partial ^{1}H NMR spectra (400 MHz, DMSO-*d*6, 298 K) of receptor $\mathbf{L}^{\mathbf{Me}}$ and obtained DMSO solution after extraction. SLE condition: 2.0 hours, 25°C, stirring rate, 1500 r/min, in DMSO with 0.8% water. The peaks within grey frameworks are assiged to free receptor, and the peaks within light purple frameworks are assigned to complexed receptors after extraction. The determined extraction efficiency are shown on the right as determined based on NMR spectra.

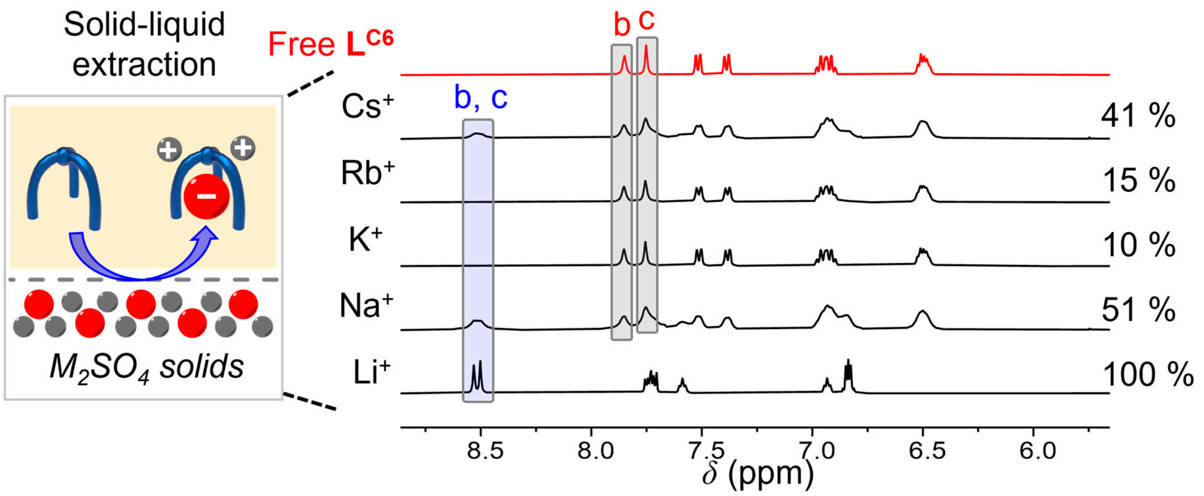

Figure S29 Stacked partial ^{1}H NMR spectra (400 MHz, DMSO-*d*6, 298 K) of receptor $\mathbf{L}^{\mathbf{C6}}$ and obtained DMSO solution after extraction. SLE condition: 2.0 hours, 25°C, stirring rate, 1500 r/min, in DMSO with 0.8% water. The peaks within grey frameworks are assiged to free receptor, and the peaks within light purple frameworks are assigned to complexed receptors after extraction. The determined extraction efficiency are shown on the right as determined based on NMR spectra.

Table S14 Concentrations of alkali cations remaining after extraction by using $\mathbf{TL^{C6}}$. SLE condition: 2 hours, 25°C, stirring rate, 1500 r/min, in DMSO with 0.8% water.

Cation	After extraction($\mathbf{TL^{C6}}$) (mM)	Extraction efficiency
Li^+	0.76±0.08	81%
Na^+	2.76±0.05	33%
K^+	3.38±0.04	17%
Rb^+	3.38±0.09	15%
Cs^+	3.82±0.01	6%

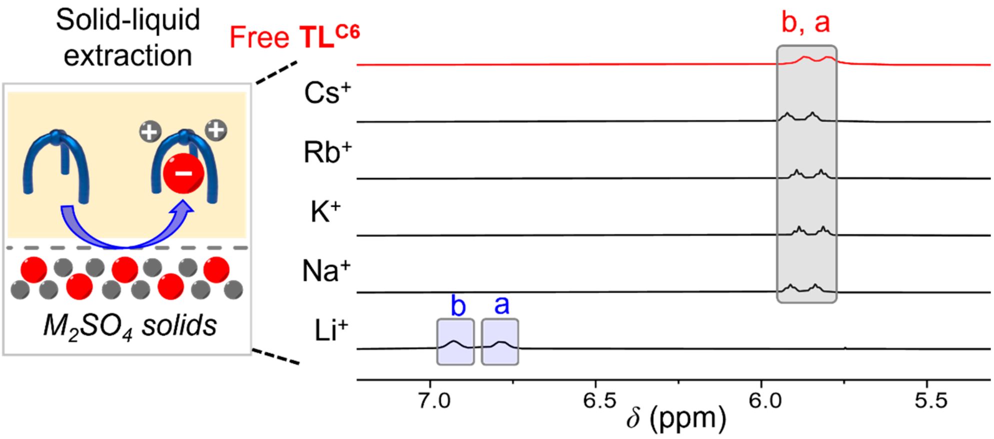

Figure S30 Stacked partial ^{1}H NMR spectra (400 MHz, DMSO-d_6, 298 K) of receptor $\mathbf{TL^{C6}}$ and obtained DMSO solution after extraction. SLE condition: 2.0 hours, 25°C, stirring rate, 1500 r/min, in DMSO with 0.8% water. The peaks within grey frameworks are assiged to free receptor, and the peaks within light purple frameworks are assigned to complexed receptors after extraction. The determined extraction efficiency are shown on the right as determined based on NMR spectra.

Table S15 Concentrations of alkali cations remaining after extraction by using $\mathbf{L^{C6}}$ with or without the presence of two equivalents of 12-crow-4 ether macrocyclese. SLE condition: 1 hour, 25°C, stirring rate, 1500 r/min, in DMSO with 0.8% water.

Cation	After extraction (mM) (12C4+Li_2SO_4)	Extraction efficiency (12C4+Li_2SO_4)	After extraction (mM) (M_2SO_4)	Extraction efficiency (M_2SO_4)
Li^+	0.35±0.05	92%	0.42±0.03	90%

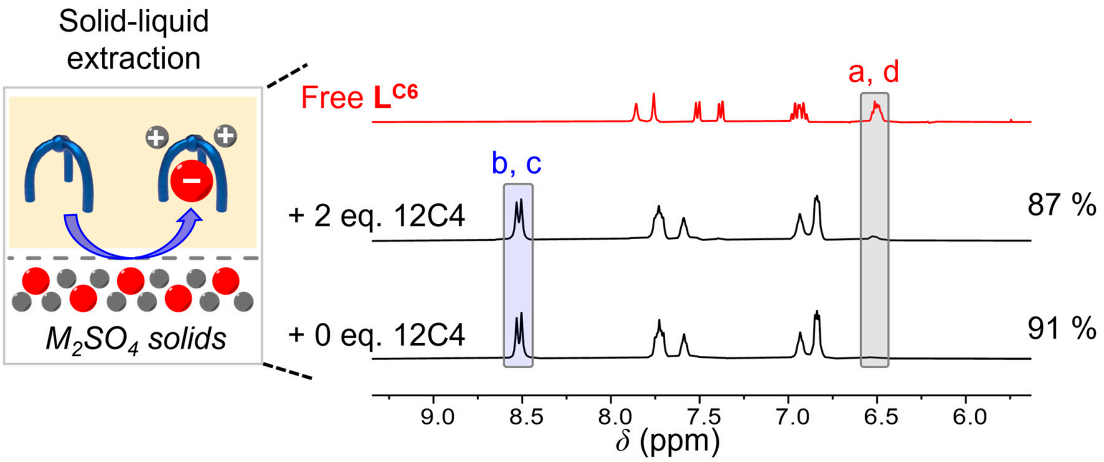

Figure S31 Stacked partial ^{1}H NMR spectra (400 MHz, DMSO-*d*6, 298 K) of receptor $\mathbf{L}^{\mathbf{C6}}$ and obtained DMSO solution with or without the presence of twp equivalents of 12-crown-4 ether macrocycles after extraction. SLE condition: 1 hour, 25°C, stirring rate, 1500 r/min, in DMSO with 0.8% water. The peaks within grey frameworks are assiged to free receptor, and the peaks within light purple frameworks are assigned to complexed receptors after extraction. The determined extraction efficiency are shown on the right as determined based on NMR spectra.

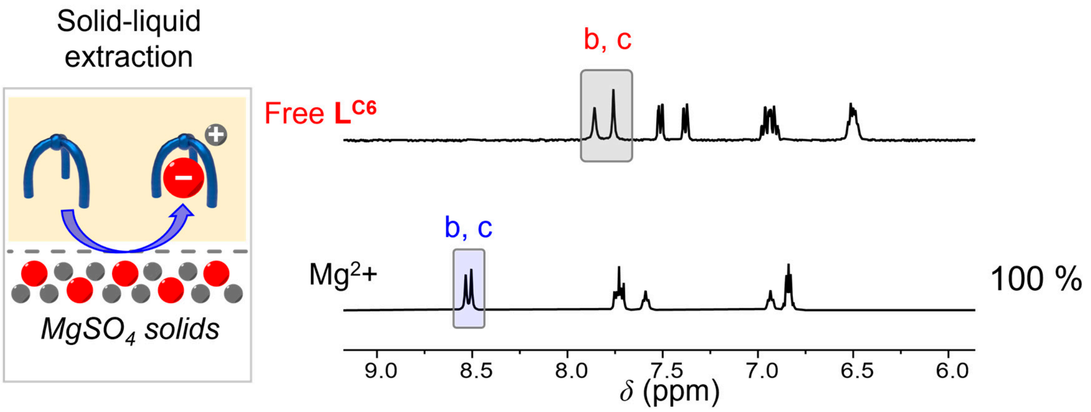

Figure S32 Stacked partial ^{1}H NMR spectra (400 MHz, DMSO-*d*6, 298 K) of receptor $\mathbf{L}^{\mathbf{C6}}$ and obtained DMSO solution with $MgSO_4$ salts after extraction. SLE condition: 2.0 hour, 25℃, stirring rate, 1500 r/min, in DMSO with 0.8% water. The determined extraction efficiency are shown on the right as determined based on NMR spectra.

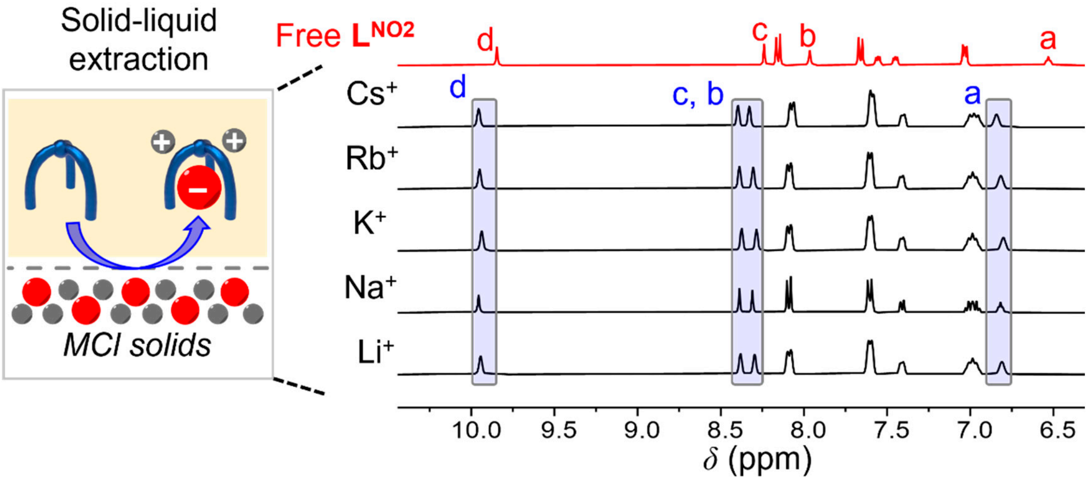

Figure S33 Stacked partial ^{1}H NMR spectra (400 MHz, DMSO-*d*6, 298 K) of receptor $\mathbf{L}^{\mathbf{NO2}}$ and obtained DMSO solution with MCl salts after extraction. SLE condition: 5.0 hour, 25°C, stirring rate, 1500 r/min, in DMSO with 0.8% water. All the salts were extracted without selectivity.

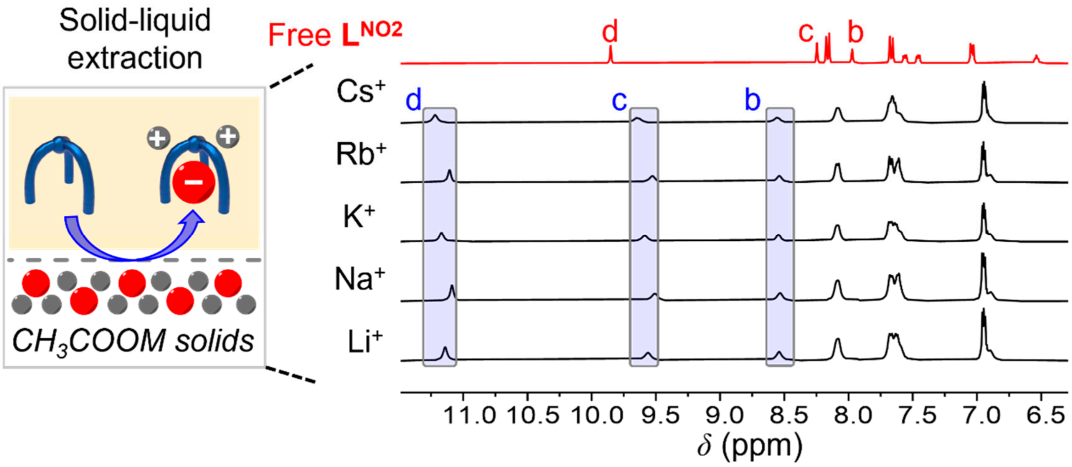

Figure S34 Stacked partial ^{1}H NMR spectra (400 MHz, DMSO-*d*6, 298 K) of receptor $\mathbf{L}^{\mathbf{NO2}}$ and obtained DMSO solution with CH_3COOM salts after extraction. SLE condition: 5.0 hour, 25°C, stirring rate, 1500 r/min, in DMSO with 0.8% water. All the salts were extracted without selectivity.

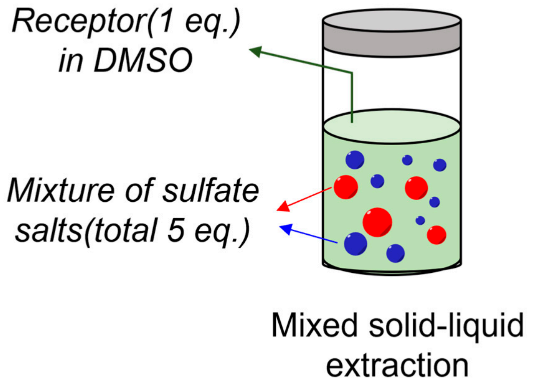

A DMSO solution (2 mL) of the extractant L^{NO2} (5 mM) was exposed to 80 µL aqueous of five targeted alkali metal sulfates (Li_2SO_4 + Na_2SO_4 + K_2SO_4 + Rb_2SO_4 + Cs_2SO_4, 5 mM respectively). Extraction was then performed by stirring for 2.0 hours under 25 °C temperature in DMSO with 0.8% water. The solution and undissolved solids are separated upon centrifugation. The organic layer is carefully collected and redissolved in DMSO-d_6 for ^{1}H NMR analyses. The bottom sediment is washed with regular DMSO (2 mL × 3) and redissolved in water (5 mL) by using a volumetric flask. An aqueous solution (0.5 mL) is taken and subjected to a 0.2 µM syringe filter, then the obtained aqueous solution is charged for ion chromatography to determine cation centration.

Table S16 Concentrations of alkali cations remaining after extraction with the presence of five alkali sulfate salts. SLE condition: 2 hour, 25℃, stirring rate, 1500 r/min, in DMSO with 0.8% water.

Cation	After extraction (L^{NO2}) (mM)	After extraction(L^{Me}) (mM)	After extraction(L^{C6}) (mM)
Li^+	2.02±0.11	2.56±0.05	2.22±0.08
Na^+	2.89±0.03	3.30±0.12	3.23±0.05
K^+	3.95±0.05	4.05±0.01	4.04±0.02
Rb^+	3.63±0.09	3.86±0.04	3.84±0.16
Cs^+	3.08±0.03	3.37±0.04	3.24±0.03

Table S17 Calculated extraction effiency based on the data shown in Table S17.

Cation	Extraction efficiency (L^{NO2})	Extraction efficiency (L^{Me})	Extraction efficiency (L^{C6})
Li^+	50%	36%	45%
Na^+	30%	20%	21%
K^+	3%	1%	1%
Rb^+	8%	3%	4%
Cs^+	24%	17%	20%

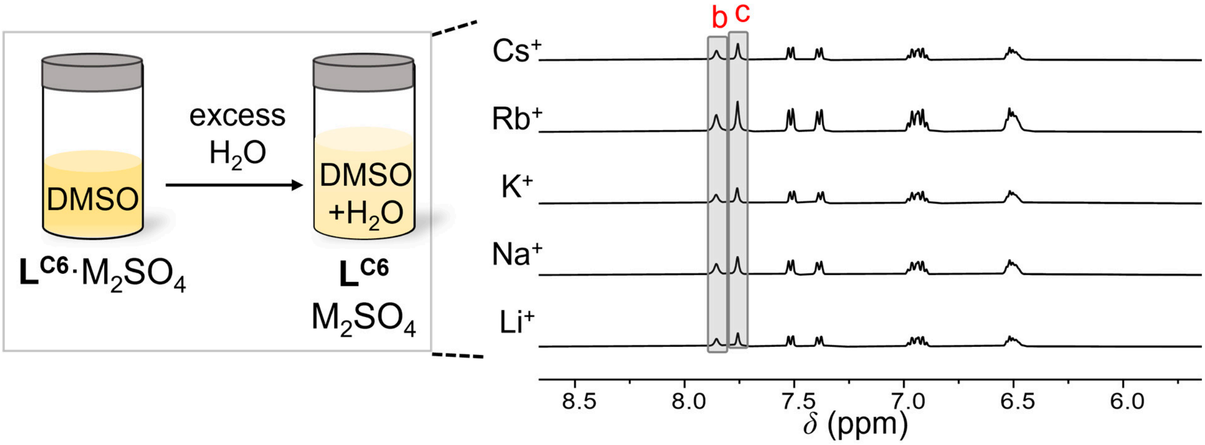

Figure S35 Stacked partial ^{1}H NMR spectra (400 MHz, DMSO-d_6, 298 K) after water wash. Based on the NMR spectra, all the receptors were recovered.

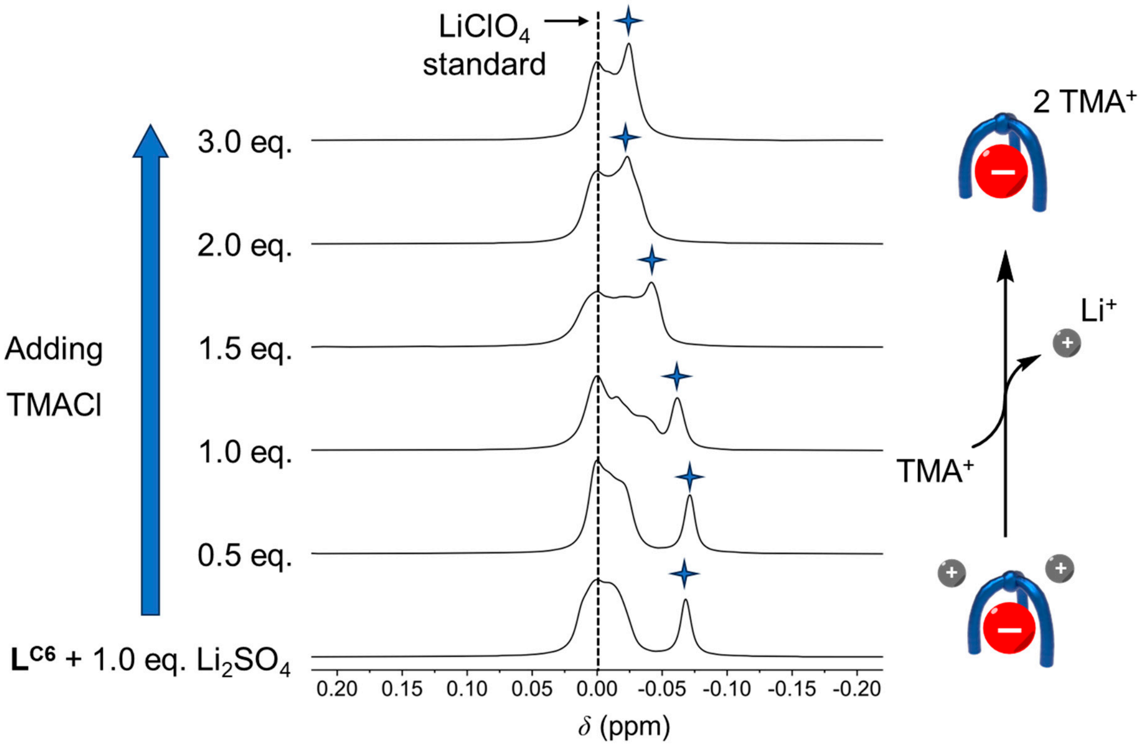

Figure *S36* Stacked ^{7}Li NMR spectra (156 MHz, 298 K, DMSO-d_6 with 3.2% H_2O, 2 mM) of Li_2SO_4 with one equivalent of $\mathbf{L^{C6}}$ receptor by adding tetramethylammonium chloride. External standard of was used, the symbol of "✦"indicates the chemical shift of Li^+.

S5. Mass Spectrometry

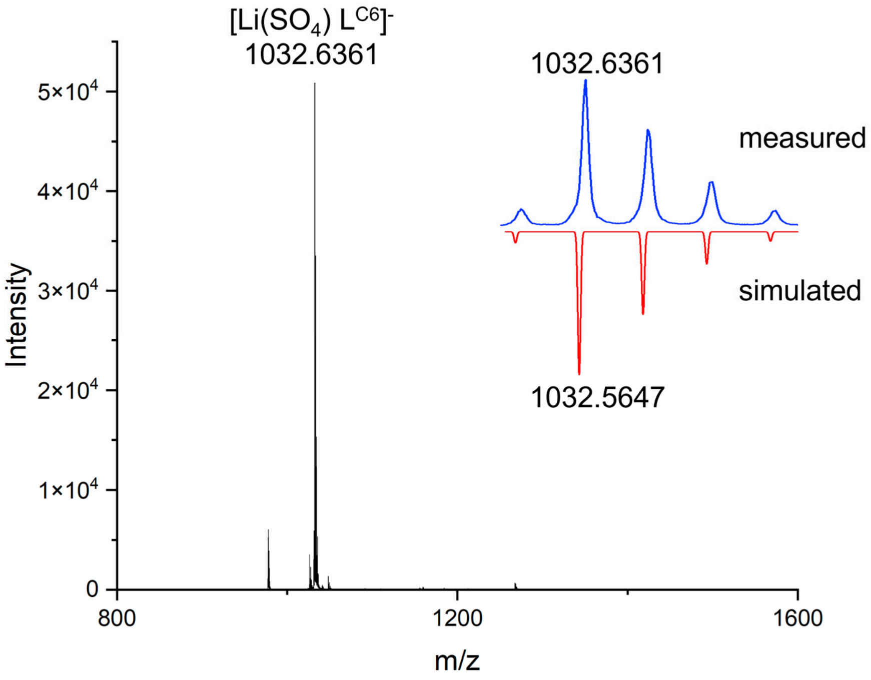

Figure S37 Obtained HR-ESI-QTOF Mass spectrum for the complex of $\mathbf{L^{C6}}$ with Li_2SO_4.

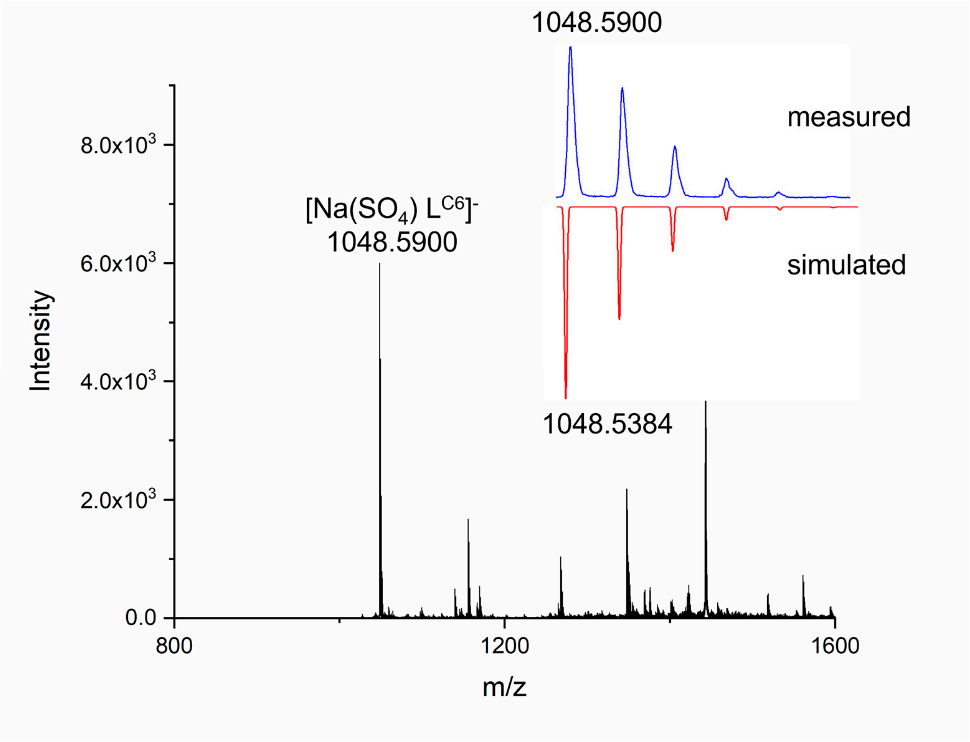

Figure S38 Obtained HR-ESI-QTOF Mass spectrum for the complex of $\mathbf{L^{C6}}$ with Na_2SO_4.

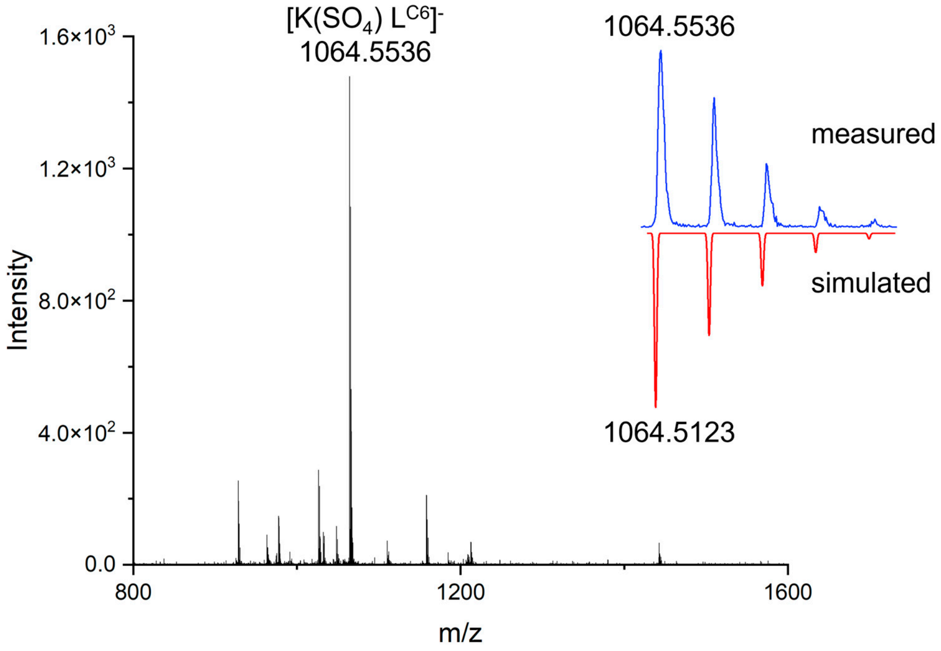

Figure S39 Obtained HR-ESI-QTOF Mass spectrum for the complex of $\mathbf{L^{C6}}$ with K_2SO_4.

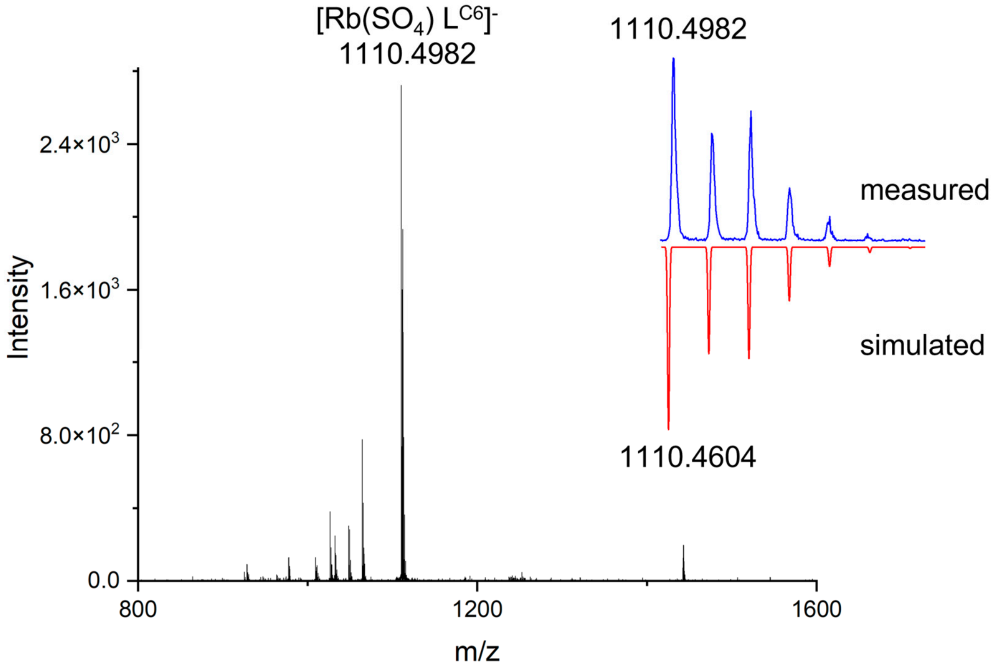

Figure S40 Obtained HR-ESI-QTOF Mass spectrum for the complex of $\mathbf{L^{C6}}$ with Rb_2SO_4.

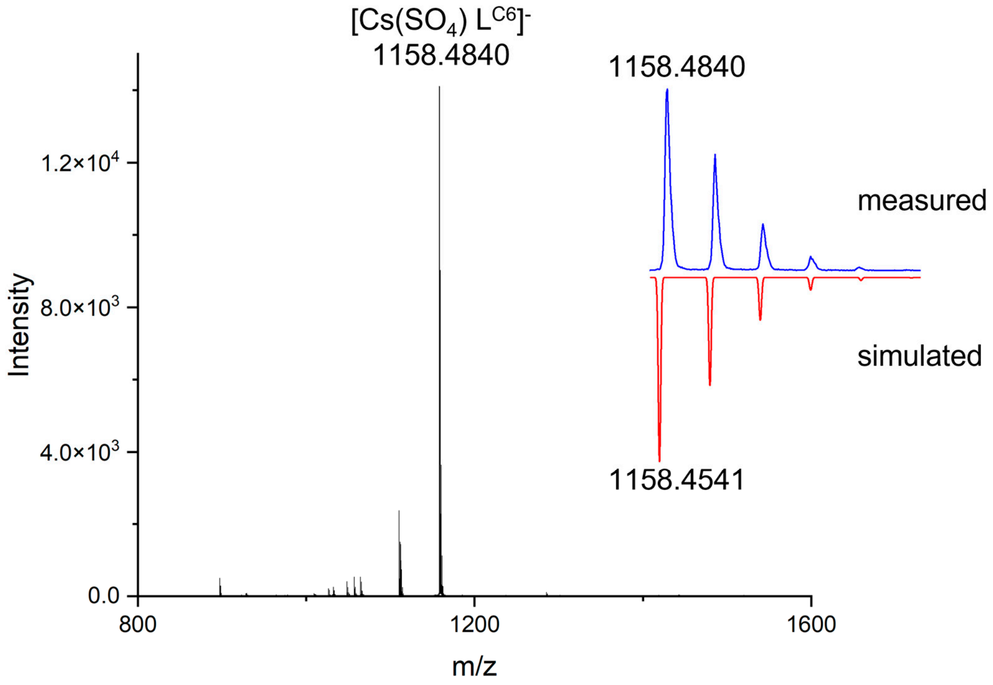

Figure S41 Obtained HR-ESI-QTOF Mass spectrum for the complex of $\mathbf{L^{C6}}$ with Cs_2SO_4.
